# How gender is associated with physical bullying and victimization across adolescent stages

**DOI:** 10.1371/journal.pone.0352450

**Published:** 2026-06-25

**Authors:** Ruining Jin, Sunqiuyu Zhang, Fengshi Ren, Xiao Wang, Tam-Tri Le

**Affiliations:** 1 Institute of Higher Education, Beijing University of Technology, Beijing, China; 2 Capital Engineering Education Development Research Base, Beijing University of Technology, Beijing, China; 3 Department of Psychology, University of Macau, Taipa, Macau, China; 4 Independent researcher, Ho Chi Minh City, Vietnam; University of Huelva: Universidad de Huelva, SPAIN

## Abstract

**Background:**

Physical bullying remains a significant form of peer aggression during adolescence, a developmental period marked by rapid bodily, psychological, and social change. Although gender differences in bullying have been widely documented, less is known about whether the association between gender and physical bullying perpetration and physical victimization varies across adolescent stages in contemporary non-WEIRD settings.

**Methods:**

This study conducted a secondary data analysis of openly available survey data from 169 school-going adolescents in India. Participants were grouped into three developmental stages: early adolescence (10–12 years, 35.5%), middle adolescence (13–15 years, 49.1%), and late adolescence (16–18 years, 14.8%). Two Bayesian regression models aided by Markov Chain Monte Carlo estimation were used to examine the association between gender and physical bullying perpetration and victimization, and to assess whether age moderated these associations.

**Findings:**

Male adolescents showed higher levels of both physical bullying perpetration and physical victimization than female adolescents. The association between male gender and physical bullying perpetration became more pronounced across later adolescent stages. By contrast, age did not clearly moderate the association between gender and physical victimization.

**Implications:**

The findings suggest that bullying prevention in school settings should pay closer attention to gendered peer dynamics, developmental stage, and school climate. In particular, school-wide and developmentally sensitive approaches may be more useful than isolated incident-based responses for addressing physical bullying in adolescent populations.

## 1. Introduction

Bullying is a pervasive issue among adolescents, with far-reaching consequences for both victims and perpetrators [1,2]. Bullying is a repeated aggressive behavior involving an interpersonal imbalance of power, where the perpetrator intentionally inflicts harm, either physically, verbally, or psychologically, on the victim [3–5]. The main types of bullying include physical (e.g., hitting, kicking, pushing), verbal (e.g., name-calling, taunting, threatening), relational (e.g., spreading rumors, social exclusion), and cyberbullying (e.g., using electronic means to harass or intimidate) [6]. Bullying behaviors are relatively prevalent in adolescents and can have severe psychological, physical, and emotional consequences for victims such as depression, anxiety, low self-esteem, fear, and absenteeism from school [7].

Physical bullying is a form of direct aggression that involves the use of physical force or intimidation to harm others [8]. Cross-national research has documented substantial between-country variation in bullying, with relatively high prevalence reported in many lower- and middle-income settings, although the pattern differs by bullying form, country context, and measurement approach [9–11]. One possible explanation is that some education systems have more developed school-wide prevention infrastructures, stronger staff training, and clearer anti-bullying policy frameworks, whereas implementation capacity and evaluated intervention evidence remain more limited in many low- and middle-income settings [12,13]. Although cyberbullying has become a major concern in recent years, physical bullying remains highly consequential for adolescent well-being. [14] found that many adolescents who experience cyberbullying also experience traditional forms of bullying, while [15] likewise showed that online and offline victimization often overlap. Physical bullying therefore remains an important and distinct dimension of adolescent peer relations rather than a phenomenon displaced by digital forms of aggression.

Regarding gender, prior research has consistently shown that boys are more likely than girls to be involved in physical forms of bullying, both as perpetrators and as victims [4,16]. Similar patterns have been reported across diverse national contexts, including the United States [17], China [18], Brazil [19], and Saudi Arabia [20]. However, gender should not be understood merely as a binary demographic characteristic that mechanically predicts aggression. Rather, feminist and queer scholarship suggests that bullying is often embedded in wider systems of gender regulation through which peer cultures and school institutions reproduce hierarchies of masculinity, femininity, and heterosexuality [21–23]. From a theoretical perspective, social learning theory remains relevant because it helps explain how students learn not only from direct experience but also from observing which behaviors are modeled, tolerated, and rewarded in their environments [24]. In this sense, teachers, alongside peers, form part of the social environment through which gendered conduct is reinforced or challenged; school-based research on adolescent masculinities has similarly emphasized the importance of positive role models and developmental programming in shaping boys’ understandings of masculinity [25], even as scholarship on male teachers cautions that such role-model effects are socially mediated rather than automatic [26]. As a result, in many school and peer contexts, boys may face stronger pressure to display toughness, dominance, and emotional restraint, whereas students who depart from normative gender expectations may become especially exposed to ridicule, harassment, or exclusion [27,28]. From this perspective, physical bullying can function not only as individual aggression but also as a socially meaningful practice through which toughness, dominance, and normative masculinity are enacted, recognized, and sometimes rewarded in school settings [29].

Prior studies on the relationship between bullying and age, however, have produced less consistent findings. Some studies suggest that bullying involvement increases with age, especially when externalizing behaviors such as aggression and defiance become more common in later stages of childhood and adolescence [30]. Other research has reported a downward trend, arguing that bullying may rise during school transitions but decline as adolescents mature cognitively and as peer norms shift [31,32,15] further showed that age-related patterns may differ across bullying types and across traditional versus online contexts. These mixed findings suggest that age may not operate as a simple predictor. Rather, adolescence is a developmental period marked by rapid bodily change, increased sensitivity to peer evaluation, status concerns, and evolving social identities [33–35], all of which may shape how gendered patterns of physical bullying emerge and are expressed.

Despite the substantial literature on bullying, the present study addresses a more specific question. Much of the existing literature has examined bullying in broad terms or has focused primarily on overall prevalence and correlates, whereas physical bullying may operate through somewhat distinct social dynamics because it is more visibly tied to bodily dominance, vulnerability, and peer-status displays [8,29]. In addition, although gender and age have both been widely studied, it remains less clear whether the commonly observed gender pattern in physical bullying and victimization varies across developmental stages of adolescence.

This question is especially relevant in the Indian context, where bullying research has expanded but remains marked by important contextual diversity [36,37]. A systematic review of bullying and victimization among adolescents in India suggests that bullying in Indian schools is shaped by multiple school, peer, and family factors rather than by individual traits alone [37]. A more recent scoping review similarly argues that bullying patterns in India are influenced by factors aligned with the broader cultural climate, underscoring the need to situate bullying within wider sociocultural contexts [36]. Therefore, the present study not only provides evidence from a non-WEIRD context, but also situates the analysis in a setting where rapid urbanization, educational change, and digitalization coexist with persistent yet evolving gender norms [38,39]. This is particularly relevant for urban school settings, because Indian schools are also sites where gender norms are reproduced, negotiated, and sometimes reshaped [40,41]. In addition, recent evidence from southern India highlights the relevance of region, school setting, and classroom context in bullying-related experiences, suggesting that urban South Indian school environments deserve closer attention rather than being treated as interchangeable with other settings [42]. Against this background, data from school-going adolescents in Chennai and Bengaluru are useful not because prior work has already fully mapped those cities’ bullying subcultures, but because they provide an opportunity to examine whether established gender patterns in physical bullying appear similarly in contemporary urban South India.

The present study contributes to the literature in three ways. First, it focuses specifically on physical bullying, rather than treating bullying as a single undifferentiated construct. Second, it examines not only gender differences but also whether age moderates the association between gender and both physical bullying and physical victimization. Third, it uses recent secondary data from school-going adolescents in Chennai and Bengaluru, thereby extending discussion beyond the predominantly Western literature and reassessing established patterns in a non-WEIRD context. Because the available dataset records gender only in male/female categories, the present study examines gendered differences as captured within a binary dataset. Thus, using secondary survey data collected in 2022 from school-going adolescents in Chennai and Bengaluru, India, the present study addresses the following research objectives:

1. To examine the association between gender and physical bullying among adolescents and to assess whether age moderates this relationship.
2. To investigate the association between gender and physical bullying victimization among adolescents and to determine whether age moderates this relationship.

## 2. Methodology

### 2.1. Materials and variables

We use secondary data from the data article “Survey data on bullying involvement among school-going adolescents in India” [43]. The data contains basic demographic information together with types and frequency of bullying involvement from 169 school-going adolescents. The participants were from two cities, Chennai and Bengaluru, in South India. The original data collection involved purposive sampling from coeducational, English-medium schools that offer both traditional and virtual classrooms. A 30–45-minute workshop was conducted in August and September 2022 to introduce the concept of bullying. The final number of legitimate participants who consented and submitted parental permission forms was 169. Further detailed information about criteria, selection process, and basic descriptive statistics is openly available online in the data article. In the ethics statement, the data collection carried out by [43] was stated to be in accordance with the Declaration of Helsinki and was reviewed and approved by the Institutional Review Board at CHRIST (Deemed to be University) (CU: RCEC/00243/11/21). Written informed consent and parental permission were acquired by [43] from all participants. Since the present study involved a secondary analysis of fully anonymized, publicly available data and did not involve new participant recruitment, contact, or data collection, no additional ethical approval was required for this analysis.

The sample includes 81 males (47.9%) and 88 females (52.1%). [43] categorized students into 3 age categories: early adolescence (10–12 years) (N = 60, 35.5%), middle adolescence (13–15 years) (N = 83, 49.1%), and late adolescence (16–18 years) (N = 25, 14.8%). In total, 53.8% (N = 91) were in middle school (grades 6–8) and 46.2% (N = 78) were in high school (grades 9–12). To enhance transparency, we have included a summary table of the available demographic characteristics (age, gender distribution, grade level, and socioeconomic status) of our sample (See Table 1).

**Table 1:** Demographic characteristics of the participants.

Variable	Sub-category	N	%
Socioeconomic Status	Low	5	3.0
	Middle	158	93.5
	High	2	1.2
	No Response	4	2.4
Gender	Male	81	47.9
	Female	88	52.1
Age	Early Adolescence (10–12)	60	35.5
	Middle Adolescence (13–15)	83	49.1
	Late Adolescence (16–18)	25	14.8
	No Response	1	0.6
Grade Level	Middle School (6–8)	91	53.8
	High School (9–12)	78	46.2

Bullying and victimization experiences were measured using the Adolescent Peer Relations Questionnaire (APRI) [44]. The questionnaire has 36 items in total and 6 subscales, with 6 items in each subscale. The APRI has been previously tested in cross-cultural contexts, proven to have high reliability and validity [45–47]. In the data article, Cronbach's alpha coefficient was reported to be 0.83 for bullying perpetration and 0.88 for bullying victimization [43]. Details about the variables used in the present study are presented in Table 2 and subsequent text below.

**Table 2:** Variable description.

Variable	Description	Value	Type
*gender*	The participant’s self-reported gender	0 is female 1 is male	Binary
*agecat*	The participant’s age category	1 is early adolescence (10–12) 2 is middle adolescence (13–15) 3 is late adolescence (16–18)	Ordinal
*pb*	Physical bullying: the degree the participant physically bullied other student(s)	Ranging from 6 to 36	Continuous
*pv*	Physical victimization: the degree the participant was physically bullied by other student(s)	Ranging from 6 to 36	Continuous

In the sample, all students self-identified as either male or female. Although the original questionnaire provided a third option for a different self-identified gender, no observations were recorded in that category; accordingly, gender was treated as a binary variable in the present analysis (0 = female, 1 = male). Age was available in the dataset as a three-level age-category variable and was treated as an ordinal predictor, coded 1 = early adolescence (10–12 years), 2 = middle adolescence (13–15 years), and 3 = late adolescence (16–18 years). This coding reflects ordered developmental stages rather than raw continuous age. Because the present study uses secondary quantitative data and does not involve direct researcher–participant interaction, our interpretation is nevertheless informed by a view of gender as socially patterned rather than biologically fixed. We therefore interpret observed male–female differences cautiously and in relation to peer culture, school context, and wider sociocultural norms, while recognizing that the binary structure of the available dataset does not capture the full range of gendered bullying experiences. The variable *pb* is the total score derived from six items assessing the extent to which the participant physically bullied other students: “Pushed or shoved a student,” “Crashed into a student on purpose as they walked by,” “Got into a physical fight with a student because I didn’t like them,” “I slapped or punched a student,” “Threw something at a student to hit them,” and “Threatened to physically hurt or harm a student.” Each item was measured on a 6-point scale: never (1), sometimes (2), once or twice a month (3), once a week (4), several times a week (5), and every day (6). The resulting sum score ranged from 6 to 36, with higher scores indicating higher levels of physical bullying. The variable *pv* is the total score derived from six items assessing the extent to which the participant was physically bullied by other students: “I was pushed or shoved,” “I was hit or kicked hard,” “Students crashed into me on purpose as they walked by,” “My property was damaged on purpose,” “Something was thrown at me to hit me,” and “I was threatened to be physically hurt or harmed.” These items used the same 6-point response format as *pb*, producing a sum score ranging from 6 to 36, with higher scores indicating higher levels of physical victimization.

Although socioeconomic status (SES) was available in the dataset, it was not included in the primary regression models because the distribution was highly concentrated in the middle-SES category, with only a very small number of observations in the low- and high-SES groups, in addition to a small amount of nonresponse. Under these conditions, including SES in the main models would likely produce unstable and difficult-to-interpret estimates and might imply a level of precision that the present sample cannot adequately support. We therefore retain SES in the descriptive profile of the sample and acknowledge this constrained treatment as a limitation and a direction for future research.

### 2.2. Analysis procedure

To address the two research objectives, we estimated two Bayesian regression models. In Model 1, the outcome variable was physical bullying (*pb*). In Model 2, the outcome variable was physical victimization (*pv*). In both models, *gender* was coded as a binary predictor (0 = female, 1 = male), and *agecat* was treated as an ordinal predictor (1 = early adolescence, 2 = middle adolescence, 3 = late adolescence). To test whether the association between *gender* and the outcome varied across developmental stages, we included an interaction term between gender and age category (*gender × agecat*).

Consistent with the focal purpose of the study, we retained a parsimonious moderation specification centered on whether the gender association differed across adolescent stages. Thus, the models were specified as follows:

Model 1 is as follows.


$$\begin{array}{c} pb\sim normal(\mu ,\sigma )(\text{1}) \end{array}$$

$$\begin{array}{c} \mu_{i}=\beta_{\text{0}}+\beta_{gender}*{gender}_{i}+\beta_{agecat*gender}*{{agecat}_{i}*gender}_{i}(\text{2}) \end{array}$$

The posterior distributions of *pb* are in the form of normal distribution. $\mu_{i}$ is the mean value of participant i’s degree of physical bullying. ${gender}_{i}$ is participant i’s gender. ${agecat}_{i}$ is the age category that participant i belonged to. Model 1 has an intercept $\beta_{\text{0}}$ and coefficients $\beta_{gender}$ and $\beta_{agecat*gender}$.

In Model 2, *pv* (physical victimization) is the outcome variable. Model 2 is as follows.


$$\begin{array}{c} pv\sim normal(\mu ,\sigma )(\text{3}) \end{array}$$

$$\begin{array}{c} \mu_{i}=\beta_{\text{0}}+\beta_{gender}*{gender}_{i}+\beta_{agecat*gender}*{{agecat}_{i}*gender}_{i}(\text{4}) \end{array}$$

Similar to Model 1, Model 2 also has an intercept $\beta_{\text{0}}$ and coefficients $\beta_{gender}$ and $\beta_{agecat*gender}$.

For statistical analysis, we used Bayesian analysis with aided Markov Chain Monte Carlo (MCMC) algorithms. The technical reasoning, analysis procedure, and result presentation follow the protocol of MCMC-aided Bayesian analytics for social sciences and psychological research [48,49].

Bayesian modeling was chosen not only because the sample size was modest, but also because it allows direct probabilistic estimation of parameter uncertainty and provides a flexible framework for evaluating interaction effects under limited-sample conditions. This was especially useful in the present study, where the central inferential question concerned moderation rather than only main effects. However, to make sure our simulated data (or “chains”) were reliable, we checked their performance using three key indicators:

1. First of all, a technique called Pareto-Smoothed Importance Sampling Leave-One-Out (PSIS-LOO) diagnostics was used [50,51]. This method checks how well our model predicts the data by looking at a value called “*k*” value for each data point. If all *k* values are below 0.5, it means our model fits well; if the *k* values are above 0.7, then this would indicate potential problems.
2. Secondly, we need to look at Effective Sample Size (*n_eff*). Think of this as the number of independent data points our simulation represents. If *n_eff* values are above 1000, it then indicates that our estimates are based on enough ‘good’ samples [52].
3. Thirdly, Gelman-Rubin Shrink Factor (*Rhat*) is another key indicator. This statistic tells us if our different simulation runs (chains) have settled to similar values. In this regard, an *Rhat* value of 1 (*Rhat* = 1) means the chains have converged and our results are stable [53,54].

In short, if a model’s *k* values are smaller than 0.5, *n_eff* is larger than 1000, and *Rhat* equal 1, then the model is deemed reliable.

Additional visual plots (trace plots, Gelman-Rubin-Brooks plots, and autocorrelation plots) are provided in the appendixes to further ensure that our MCMC simulation worked properly. To allow replication of our study, here are the setups: the analysis was conducted using the *bayesvl* package in R [55], with R version 4.2.0, uninformative priors (for minimizing subjective influences), 5000 iterations (including 2000 warm-up iterations), and 4 chains.

## 3. Results

### 3.1. Model 1

**Fig 1:**
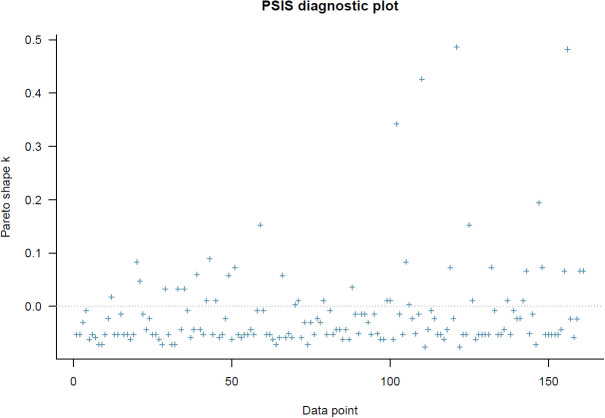
Model 1’s PSIS diagnostic plot.

The PSIS diagnostic plot for Model 1 (Fig 1) shows that the first indicator mentioned above--*k* values are lower than 0.5, and there are no values that are higher than 0.7. This indicates that Model 1’s predictions are reliable.

The other two indicators *n_eff* and *Rhat* mentioned above can be verified in Table 3 below, as *n_eff* > 1000 and *Rhat* = 1. These indicators show that the Markov chains had good convergence for Model 1, meaning that the model is reliable.

**Table 3:** Model 1’s simulated posteriors.

Parameters	Mean (M)	Standard deviation (S)	*95% CrI*	*n_eff*	*Rhat*
Constant	7.58	0.37	(6.89, 8.30)	8942	1
*gender*	0.75	1.09	(−1.35, 2.82)	6227	1
*agecat_gender*	0.57	0.55	(−0.50, 1.63)	6591	1

*Note. CrI = credible interval. The 95% CrI was calculated using the 2.5% and 97.5% posterior quantiles.*

**Fig 2:**
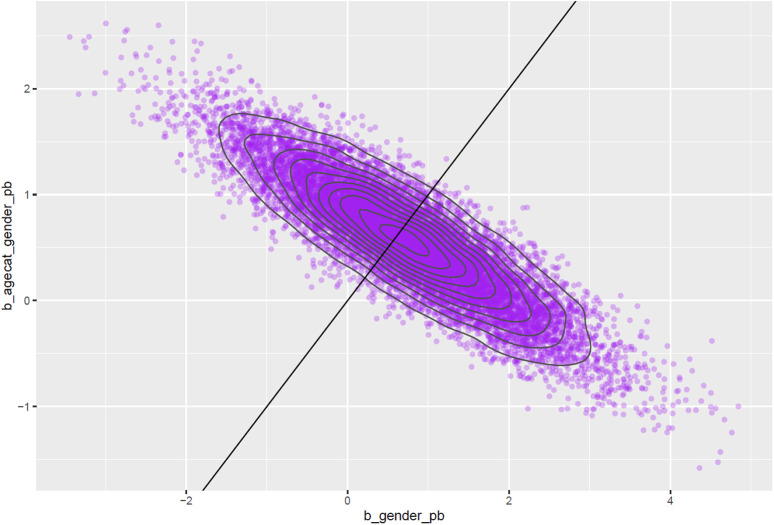
Model 1’s posterior distributions.

Estimated posterior coefficients (see Table 3) show that male gender is associated with a higher degree of physical bullying (M = 0.75, SD = 1.09). Fig 2 presents the joint posterior distribution of the gender coefficient and the gender × agecat coefficient. The posterior mass for the gender coefficient was concentrated predominantly in the positive region, suggesting a positive tendency in the association between male gender and physical bullying, although the 95% CrI included zero. The posterior mass for the gender × age-category interaction term was also mainly positive, but more dispersed. Substantively, this suggests that the estimated male–female difference in physical bullying may become larger across later adolescent stages. In practical terms, male adolescents were estimated to report higher physical bullying than female adolescents, and this estimated gap appeared somewhat larger from early to middle and late adolescence. However, because the 95% CrI for the interaction term included zero, this pattern should be interpreted as a suggestive tendency rather than as strong evidence of a definitive moderation effect.

### 3.2. Model 2

**Fig 3:**
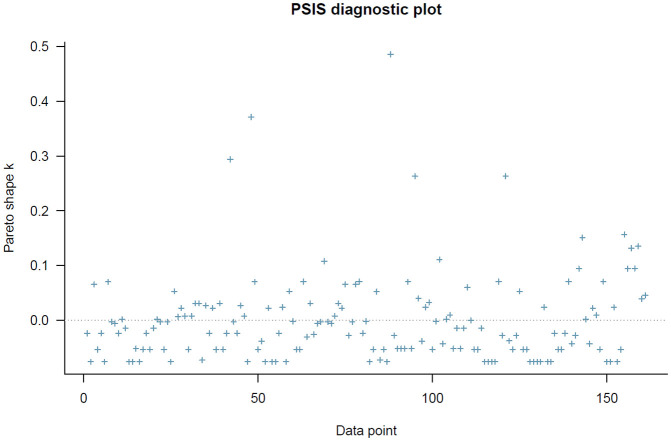
Model 2’s PSIS diagnostic plot.

The PSIS diagnostic plot for Model 2 (Fig 3) also shows that all *k* values are lower than 0.5, and no values are larger than 0.7, which mean that the first indicator (*k* value) suggests Model 2 is reliable.

The second and third indicators for Model 2 also suggest the model’s reliability is good, as all *n_eff* values are greater than 1000, and all *Rhat* values equal 1 (see Table 4).

**Table 4:** Model 2’s simulated posteriors.

Parameters	Mean (M)	Standard deviation (S)	*95% CrI*	n_eff	Rhat
Constant	8.34	0.49	(7.39, 9.31)	8844	1
*gender*	2.51	1.45	(−0.45, 5.40)	5980	1
*agecat_gender*	0.12	0.74	(−1.33, 1.62)	5929	1

*Note. CrI = credible interval. The 95% CrI was calculated using the 2.5% and 97.5% posterior quantiles.*

**Fig 4:**
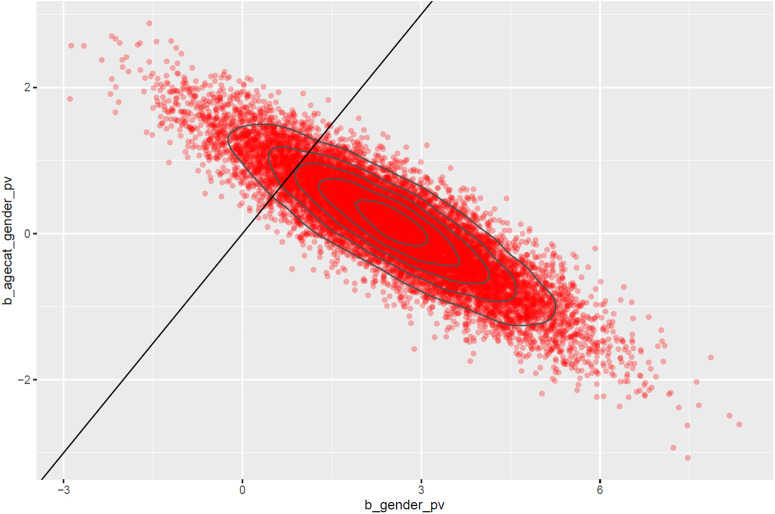
Model 2’s posterior distributions.

Estimated posterior coefficients (see Table 4) show that male gender is associated with a higher degree of physical victimization (M = 2.51, SD = 1.45). Fig 4 presents the joint posterior distribution of the gender coefficient and the gender × agecat coefficient. The posterior mass for the gender coefficient was concentrated predominantly in the positive region, suggesting a positive tendency in the association between male gender and physical victimization. By contrast, the posterior mass for the gender × age-category interaction term was much more diffuse and remained close to the weaker-effect region, indicating substantial uncertainty regarding age-based moderation. Substantively, this means that although male adolescents were estimated to report higher physical victimization than female adolescents, the estimated male–female difference in victimization did not clearly increase or decrease across early, middle, and late adolescence. Thus, unlike physical bullying perpetration, the gendered pattern of physical victimization appeared less sensitive to adolescent stage in this sample.

## 4. Discussion

The analysis results show that male adolescents are more likely to report physical bullying and physical victimization than female adolescents. In addition, the results suggest that the positive association between male gender and physical bullying becomes more pronounced across later adolescent stages in this sample, whereas the association between gender and physical victimization does not show a comparably clear age-based moderation pattern. Because the present study is based on secondary cross-sectional data, these findings should be interpreted as associations rather than as direct evidence of causal developmental mechanisms.

### 4.1. Discussion of RQ1

In the data article from which the present study retrieved the dataset [43], the authors presented a brief table of multiple linear regression across variables, but did not include the gender factor and did not find any significant correlation between age and physical bullying as well as physical victimization. The present study explored the influence of gender and the moderating effect of age on such aspects of bullying experiences. Our results on the gender differences in physical bullying and victimization are in alignment with prior findings [4,16,17,56]. One possible explanation is that gendered socialization processes, where society expects boys to be tough, dominant, and physically assertive, while girls are often encouraged to be nurturing and conflict‐avoidant, play a crucial role in shaping bullying behaviors [28,29]. For instance, research has shown that traditional masculinity norms are linked to higher physical aggression in boys because they valorize strength and assertiveness [29].

The age moderation observed in Model 1 suggests that the association between male gender and physical bullying may become more pronounced across later adolescent stages in this sample. One plausible interpretation is that, as adolescents grow older, peer status concerns, susceptibility to peer influence, and school-based expectations around masculinity may become more salient, making physical forms of dominance more socially meaningful for some boys [57,58]. This interpretation is also broadly consistent with social learning theory and with scholarship linking hegemonic masculinity to bullying behavior [24,29].

### 4.2. Discussion of RQ2

The analysis result of Model 2 suggests that while male adolescents reported higher physical victimization than female adolescents, the association between gender and physical victimization did not vary clearly across adolescent stages. In substantive terms, this means that the data support a gender difference in physical victimization, but do not provide strong evidence that this gender gap systematically widens or narrows across the three age categories included in the sample.

One possible interpretation is that the processes shaping physical victimization differ from those shaping bullying. Whereas physical bullying may be more closely tied to status performance and gendered behavioral expectations [29,58], physical victimization may depend on a broader set of contextual factors, including peer networks, school climate, situational opportunity, and local interaction patterns that were not measured in the present dataset [59,60]. For this reason, the non-significant moderation pattern in Model 2 should not be overread as evidence that age is irrelevant to victimization. Rather, it suggests that age did not clearly alter the gender–victimization association in this sample, as age itself was operationalized here through broad developmental stages. Although broader gender norms may be relevant to the observed pattern, the present dataset does not directly measure religious beliefs, cultural ideology, target sex, or specific peer-group motivations. Accordingly, the findings should not be interpreted as evidence for any particular cultural or gender-normative mechanism. These remain plausible contextual interpretations, but they cannot be established on the basis of the present data and should be tested more directly in future research.

It is also worth noting that physical bullying should be interpreted in relation to contemporary digital forms of peer aggression. Prior research has shown that traditional bullying and cyberbullying often overlap rather than occurring in isolation [14,15]. Thus, although the present study focused specifically on physical bullying, the findings should not be read as implying that offline aggression is disconnected from adolescents’ broader online–offline social worlds. Instead, the persistence of gendered patterns in physical bullying suggests that embodied, face-to-face aggression remains salient even in an era when digital interaction is central to adolescent social life. Future research could examine whether the age and gender pattern observed here is reproduced across cyberbullying or hybrid forms of bullying.

### 4.3. Implications

Based on the findings in the present study, there are several implications for school policy and intervention, especially in Asian contexts and regions of emerging economies. It should be noted that in practice, students do not always experience teachers’ anti-bullying responses as helpful and evidence suggests a gap between teacher intentions or self-reported intervention tendencies and students’ perceptions of how bullying is actually handled [61,62]. Thus, more holistic and long-term approaches integrated into the educational system are needed instead of fragmented incident-by-incident responses.

Given that the present study focuses on physical bullying, one practical implication is that schools may benefit from structured environments that channel peer status concerns, conflict, and competition into supervised and prosocial forms of interaction. The findings should not be interpreted as evidence that developmental stage itself causes bullying. Rather, the results suggest that school environments may shape how gendered expectations, status competition, and emotion regulation are expressed in peer relations. In this sense, prevention efforts should focus on modifiable school-level conditions, including peer norms, adult intervention consistency, classroom climate, and opportunities for nonviolent conflict resolution.

Specific measures integrated into the physical education curriculum may be useful when they are explicitly designed for bullying prevention. However, the broader implication is not limited to physical education alone. Updated evidence from school-based intervention research suggests that anti-bullying efforts are generally more effective when they are embedded in whole-school prevention frameworks, supported by clear policies, classroom expectations, peer involvement, and parent communication [12]. Universal social-emotional learning programs may also be relevant, because they improve emotion regulation, interpersonal competence, and behavioral adjustment in ways that can support bullying prevention more broadly [63].

In the present study, male adolescents showed higher involvement in both physical bullying perpetration and physical victimization, and the Introduction framed these patterns partly through gendered socialization and social learning processes. From this perspective, school and family contexts may be important sites for reinforcing respectful forms of peer interaction, emotional regulation, and nonviolent responses to conflict. Research on school-based bullying prevention further suggests that interventions are more effective when they are integrated into broader school-wide efforts rather than limited to reactive responses to individual incidents [64,65]. Accordingly, prevention strategies may benefit from combining clear adult responses, supportive classroom climates, and opportunities for students to develop interpersonal and self-regulatory skills. In the context of the present study, this suggests that implementation in urban Indian schools may require more than generic anti-bullying messaging. It may also require school-level conditions that support consistency in adult intervention, emotionally safe classroom climates, and developmentally appropriate attention to peer interaction and gendered expectations. Because the present sample was relatively small and drawn from specific urban settings in South India, these implications should be considered preliminary rather than universally generalizable.

### 4.4. Limitations

In this subsection, we present the limitations of the present study as well as recommended directions for further research. First, the sample comprised 169 school-going adolescents from two major metropolitan areas in South India. Although these cities are significant urban centers, the sample may not fully represent the broader adolescent population in these areas, especially given variation in socioeconomic status, school type, and urban–rural context. Future research should consider more diverse and stratified samples to better capture these subpopulations. This point is especially important given that the original workshop reached a much larger pool of students, but only a minority ultimately participated in the final dataset.

Second, among the 700 students who participated in the initial workshop, only 169, approximately 24%, submitted parental permission forms and were included in the final dataset. This low consent return rate raises an important risk of self-selection bias. It is possible that adolescents whose families were less engaged with school communication, had lower trust in institutional procedures, or were dealing with more sensitive bullying-related experiences may have been less likely to return parental consent forms. Families of highly vulnerable adolescents, or those whose children had a history of bullying perpetration or victimization, may also have been reluctant to participate because of stigma, discomfort, or fear of disclosure. If this occurred, the analytic sample may underrepresent students with more severe bullying involvement, thereby compressing the observed range of bullying and victimization scores and potentially attenuating the estimated associations. The findings should therefore be interpreted as patterns among consenting participants rather than as fully representative estimates for the broader student population.

Third, the data used in this study are cross-sectional and were collected during the post-COVID-19 period. While schools had largely resumed in-person classes at the time of data collection, lingering effects of the pandemic on peer interaction and school routines may still have shaped adolescents’ bullying experiences. Longitudinal research would be necessary to confirm the developmental pattern suggested by the present findings and to evaluate temporal ordering more rigorously.

Fourth, although SES was available in the dataset, it was not incorporated into the primary regression models because the distribution was highly imbalanced, with the overwhelming majority of participants concentrated in the middle-SES category and only very small numbers in the low- and high-SES groups. As a result, the present study cannot fully assess whether socioeconomic position conditions the observed gendered patterns of physical bullying and victimization. Future research with more balanced SES variation should examine this issue more directly.

Another limitation of our study is that the dataset categorizes gender as binary (male/female), as no participants identified themselves outside of this binary gender framework. Consequently, our findings regarding gender differences in physical bullying and victimization are constrained to this binary classification and may not capture the full spectrum of gender identities and their associated experiences. This limitation is not only methodological but also analytic: recent research suggests that gender-diverse adolescents often face disproportionately high levels of bullying and related victimization, meaning that an especially vulnerable group falls outside the scope of the present dataset. Future research should therefore incorporate more inclusive gender measures and explicitly examine how bullying dynamics differ across transgender, nonbinary, and other gender-diverse youth.

An additional limitation of the present study is the exclusive reliance on self-reported measures for assessing bullying behaviors. Self-reports are susceptible to social desirability bias, whereby participants may underreport their own bullying behavior or reinterpret victimization experiences in socially shaped ways. Future studies should aim to reduce this limitation by incorporating multiple informants, such as teachers, peers, or parents, or by using observational and mixed-method approaches where feasible.

It is also important to note that while bullying manifests in multiple forms, such as physical, relational, verbal, and cyberbullying, the present study exclusively focuses on physical bullying. Prior research has demonstrated that gender differences may vary across bullying type, with physical bullying typically more common among boys and relational bullying often more common among girls [27]. In light of the contemporary overlap between traditional and digital bullying, future work should also examine whether the patterns identified here extend to cyberbullying or mixed online–offline victimization. A broader multi-form approach would provide a more comprehensive understanding of gendered bullying dynamics.

## Appendices

**Fig 5:**
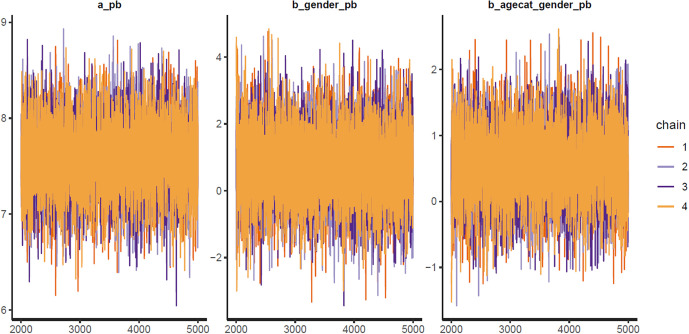
Model 1’s trace plots.

**Fig 6:**
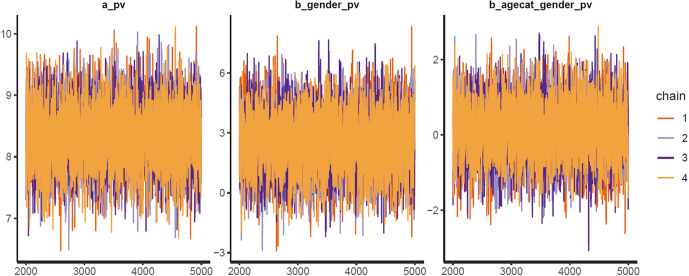
Model 1’s Gelman-Rubin-Brooks plots.

**Fig 7:**
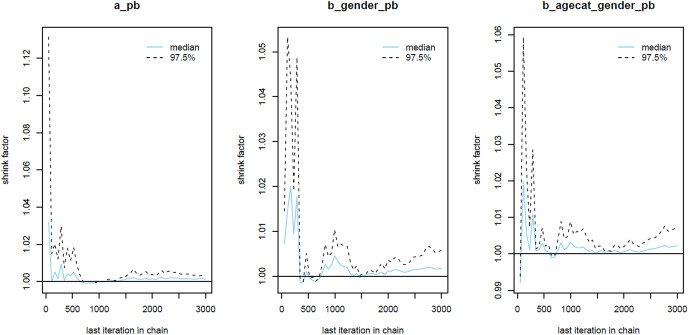
Model 1’s autocorrelation plots.

**Fig 8:**
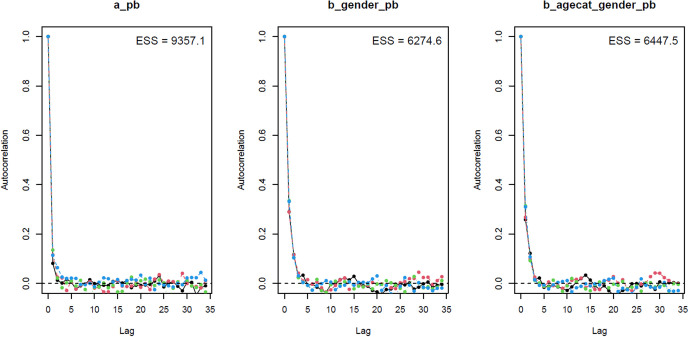
Model 2’s trace plots.

**Fig 9:**
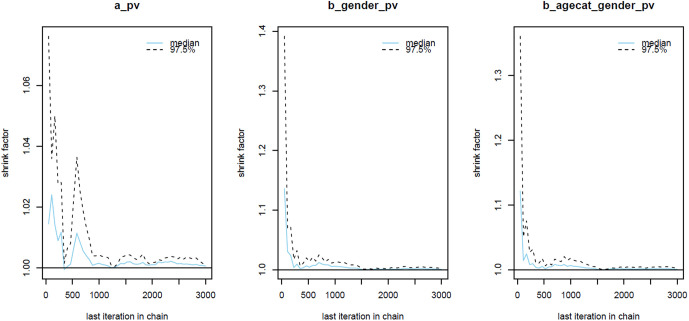
Model 2’s Gelman-Rubin-Brooks plots.

**Fig 10:**
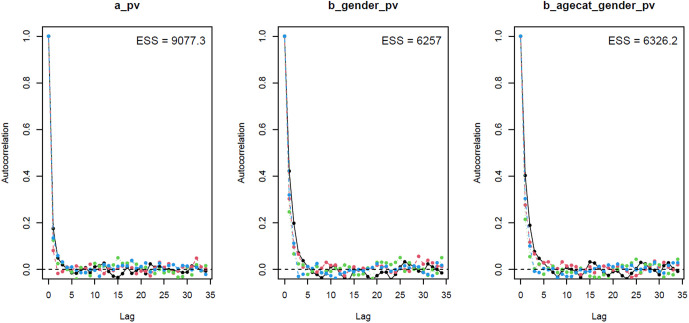
Model 2’s autocorrelation plots.

In Model 1’s trace plots (Fig 5), the colored lines represent the Markov chains. In each plot, the chains fluctuate around a central equilibrium after 2,000 iterations (warmup period), suggesting stationary qualities. Additionally, the Gelman-Rubin-Brooks plots show that *Rhat* values dropped to 1 during the warm-up period (Fig 6). The autocorrelation plots show a quick elimination of problematic autocorrelation among simulated data points within the MCMC processes (Fig 7). The trace plots (Fig 8), Gelman-Rubin-Brooks plots (Fig 9), and autocorrelation plots (Fig 10) all indicate that good Markov properties were achieved for Model 2.
