# Supplementary material for: How gender is associated with physical bullying and victimization across adolescent stages
Source: PLoS One. 2026 Jun 25;21(6):e0352450. doi: 10.1371/journal.pone.0352450 (PMC13298761; doi:10.1371/journal.pone.0352450)
Supplement: Attachment — Submitted filename: 20260415 reviewer 2.docx [file pone.0352450.s002.docx]

**Letter of responses to Reviewer 2**

Dear Reviewer,

Thank you very much for spending a great amount of time and effort reviewing our manuscript. Your detailed comments have helped us improve the quality of our paper.

In the revised manuscript, the revised parts are highlighted in yellow. In this letter, *your comments are in italic*, and **our answers are in bold**.

*****

*Reviewer #2: 
This manuscript addresses an important and timely topic, and the authors are to be commended for conducting their study in a non-WEIRD region, making a valuable contribution to a more globally diverse research landscape. The inclusion of gender and age differences as well as their interactions is a particular strength, as it opens up meaningful possibilities for the development of targeted intervention strategies. The study's commitment to open science principles in terms of data and analysis transparency is also very much appreciated.*
**We thank you very much for your recognition and kind words.** *Suggestions for revision

General: Socioeconomic status (SES)
Since SES was collected as a variable, readers may wonder about its potential role in the observed patterns of bullying aggression and victimization. It would be helpful if the authors could either incorporate SES more explicitly into the analysis and discussion, or briefly explain their reasoning for not doing so.*

**We sincerely thank the reviewer for this helpful comment. We agree that socioeconomic status (SES) may be relevant to patterns of bullying perpetration and victimization, and we appreciate the reviewer’s suggestion to clarify how this variable was handled in the present study.**

**In response, we revised the manuscript to explain more explicitly why SES was not incorporated into the primary regression models. Although SES was available in the dataset, its distribution was highly imbalanced, with the overwhelming majority of participants concentrated in the middle-SES category, only very small numbers in the low- and high-SES groups, and a small amount of nonresponse. Under these conditions, we were concerned that including SES in the main models would produce unstable and difficult-to-interpret estimates and might suggest a level of precision that the present sample could not adequately support. We therefore retained SES in the descriptive profile of the sample, clarified this rationale in the Methods section, and acknowledged the constrained treatment of SES more explicitly in the Limitations section. We now also note that future research with more balanced SES variation should examine whether socioeconomic position conditions the observed gendered patterns of physical bullying and victimization more directly.**

*…*

Although socioeconomic status (SES) was available in the dataset, it was not included in the primary regression models because the distribution was highly concentrated in the middle-SES category, with only a very small number of observations in the low- and high-SES groups, in addition to a small amount of nonresponse. Under these conditions, including SES in the main models would likely produce unstable and difficult-to-interpret estimates and might imply a level of precision that the present sample cannot adequately support. We therefore retain SES in the descriptive profile of the sample and acknowledge this constrained treatment as a limitation and a direction for future research.

*…*

Fourth, although SES was available in the dataset, it was not incorporated into the primary regression models because the distribution was highly imbalanced, with the overwhelming majority of participants concentrated in the middle-SES category and only very small numbers in the low- and high-SES groups. As a result, the present study cannot fully assess whether socioeconomic position conditions the observed gendered patterns of physical bullying and victimization. Future research with more balanced SES variation should examine this issue more directly.

*…*

*Introduction: Regional and contextual specificity
The manuscript would benefit from a richer engagement with the specific cultural, social, and institutional context of the study setting. This might include regional particularities in school norms, local social norms around bullying, and potentially the influence of religion on social behavior. Some of this context does appear in the discussion, which is great — the authors might consider introducing elements of it already in the introduction to help orient the reader from the outset.*
**We sincerely thank the reviewer for this valuable comment. We agree that the earlier version of the manuscript did not provide sufficient contextualization of the specific cultural, social, and institutional setting of the study. This suggestion was very helpful in prompting us to strengthen the Introduction and orient the reader more clearly from the outset.**

**In response, we substantially revised the Introduction to engage more directly with the Indian and urban South Indian context. Specifically, we now draw on recent review literature on bullying in India to show that bullying patterns are shaped by school, peer, and wider sociocultural conditions rather than by individual traits alone. We also added discussion of rapid urbanization, educational change, and evolving gender norms, as well as literature highlighting Indian schools as sites where gender norms are reproduced, negotiated, and sometimes reshaped. In addition, we incorporated more explicit rationale for focusing on Chennai and Bengaluru by noting that recent evidence from southern India points to the importance of region, school setting, and classroom context in bullying-related experiences. At the same time, we approached this contextualization cautiously and avoided attributing the findings to specific religious or cultural mechanisms that were not directly measured in the dataset. We believe these revisions have made the Introduction more grounded, better oriented to the study setting, and more responsive to the reviewer’s suggestion.**

**…**

This question is especially relevant in the Indian context, where bullying research has expanded but remains marked by important contextual diversity[36,37]. A systematic review of bullying and victimization among adolescents in India suggests that bullying in Indian schools is shaped by multiple school, peer, and family factors rather than by individual traits alone [37]. A more recent scoping review similarly argues that bullying patterns in India are influenced by factors aligned with the broader cultural climate, underscoring the need to situate bullying within wider sociocultural contexts [36]. Therefore, the present study not only provides evidence from a non-WEIRD context, but also situates the analysis in a setting where rapid urbanization, educational change, and digitalization coexist with persistent yet evolving gender norms [38,39]. This is particularly relevant for urban school settings, because Indian schools are also sites where gender norms are reproduced, negotiated, and sometimes reshaped [40,41]. In addition, recent evidence from southern India highlights the relevance of region, school setting, and classroom context in bullying-related experiences, suggesting that urban South Indian school environments deserve closer attention rather than being treated as interchangeable with other settings [42]. Against this background, data from school-going adolescents in Chennai and Bengaluru are useful not because prior work has already fully mapped those cities’ bullying subcultures, but because they provide an opportunity to examine whether established gender patterns in physical bullying appear similarly in contemporary urban South India.

**…**

*Introduction: Theoretical depth on gender norms
The section on gender norms has good foundations, but could be enriched by engaging more deeply with relevant theoretical frameworks. At present, the discussion focuses primarily on norms around normatively "masculine" behavior, and there is room to broaden this. For example, drawing on Bandura's social learning theory, the role of behavioral modeling by (male) teachers could be explored more explicitly. It would also be valuable to consider the developmental-psychological and physiological challenges that adolescent boys navigate during puberty, and how these may shape their social roles within the school context in ways that could contribute to bullying dynamics. Framing the phenomenon within a biopsychosocial model could provide a useful integrative structure for these considerations.*
**We sincerely thank the reviewer for this thoughtful and constructive suggestion. We agree that the earlier version of the manuscript did not engage deeply enough with the theoretical framing of gender norms and adolescent development. This comment was especially valuable in helping us strengthen the conceptual foundation of the Introduction and move beyond a narrower focus on normatively masculine behavior alone.**

**In response, we substantially revised the Introduction to broaden and deepen the theoretical discussion. First, we now engage more explicitly with Bandura’s social learning theory to clarify that bullying-related behavior may be shaped not only through peer interaction but also through the broader social environment in which behavior is modeled, tolerated, and reinforced. In this context, we added a more explicit discussion of teachers, alongside peers, as part of the social environment through which gendered conduct may be reinforced or challenged. Second, we expanded the developmental framing of adolescence by emphasizing that this period involves rapid bodily change, increased sensitivity to peer evaluation, status concerns, and evolving social identities, all of which may shape how gendered patterns of physical bullying emerge and are expressed in school settings. Third, while we did not adopt a formal biopsychosocial model as the core analytic framework of the manuscript, we revised the Introduction to reflect a broader developmental and contextual perspective in which biological, psychological, and social processes are treated as relevant background for interpreting adolescent bullying dynamics more cautiously and more comprehensively.**

**We are grateful for this suggestion, as it significantly improved the conceptual depth and explanatory coherence of the manuscript. The revised text is reproduced below:**

Regarding gender, prior research has consistently shown that boys are more likely than girls to be involved in physical forms of bullying, both as perpetrators and as victims [4,16]. Similar patterns have been reported across diverse national contexts, including the United States [17], China [18], Brazil [19], and Saudi Arabia [20]. However, gender should not be understood merely as a binary demographic characteristic that mechanically predicts aggression. Rather, feminist and queer scholarship suggests that bullying is often embedded in wider systems of gender regulation through which peer cultures and school institutions reproduce hierarchies of masculinity, femininity, and heterosexuality [21–23]. From a theoretical perspective, social learning theory remains relevant because it helps explain how students learn not only from direct experience but also from observing which behaviors are modeled, tolerated, and rewarded in their environments [24]. In this sense, teachers, alongside peers, form part of the social environment through which gendered conduct is reinforced or challenged; school-based research on adolescent masculinities has similarly emphasized the importance of positive role models and developmental programming in shaping boys’ understandings of masculinity [25], even as scholarship on male teachers cautions that such role-model effects are socially mediated rather than automatic [26]. As a result, in many school and peer contexts, boys may face stronger pressure to display toughness, dominance, and emotional restraint, whereas students who depart from normative gender expectations may become especially exposed to ridicule, harassment, or exclusion [27,28]. From this perspective, physical bullying can function not only as individual aggression but also as a socially meaningful practice through which toughness, dominance, and normative masculinity are enacted, recognized, and sometimes rewarded in school settings [29].

Prior studies on the relationship between bullying and age, however, have produced less consistent findings. Some studies suggest that bullying involvement increases with age, especially when externalizing behaviors such as aggression and defiance become more common in later stages of childhood and adolescence [30]. Other research has reported a downward trend, arguing that bullying may rise during school transitions but decline as adolescents mature cognitively and as peer norms shift [31,32]. [15] further showed that age-related patterns may differ across bullying types and across traditional versus online contexts. These mixed findings suggest that age may not operate as a simple predictor. Rather, adolescence is a developmental period marked by rapid bodily change, increased sensitivity to peer evaluation, status concerns, and evolving social identities[33–35], all of which may shape how gendered patterns of physical bullying emerge and are expressed.

**…**

*Introduction/Methods: Positionality and contextualization
A brief reflexive statement on the positionality of the research team would be a welcome addition. It would help readers understand the perspective from which masculinity and gender norms are conceptualized, and how the authors' own standpoints may have informed the framing of the research questions and the interpretation of findings.*
**We sincerely thank the reviewer for this thoughtful suggestion. We agree that, even in a secondary quantitative study, it is helpful to clarify the interpretive perspective from which masculinity and gender norms are conceptualized. At the same time, because the present study relies on anonymized, publicly available secondary data and does not involve direct researcher–participant interaction, we considered a brief reflexive statement to be more proportionate than a longer qualitative-style positionality section. In response, we added a short statement in the Methods section clarifying that our interpretation is informed by a view of gender as socially patterned rather than biologically fixed, and that observed male–female differences are interpreted cautiously in relation to peer culture, school context, and wider sociocultural norms. We also note explicitly that the binary structure of the available dataset constrains the range of gendered experiences that can be captured. We are grateful for this suggestion, as it helped us make our interpretive standpoint more transparent without extending beyond what is appropriate for the present study design.**

**…**

Because the present study uses secondary quantitative data and does not involve direct researcher–participant interaction, our interpretation is nevertheless informed by a view of gender as socially patterned rather than biologically fixed. We therefore interpret observed male–female differences cautiously and in relation to peer culture, school context, and wider sociocultural norms, while recognizing that the binary structure of the available dataset does not capture the full range of gendered bullying experiences.

*…
Discussion: Digital forms of bullying
It would be worth reflecting more explicitly in the discussion on how the findings relate to contemporary digital forms of bullying. Given how central online spaces have become to adolescent social life, even a brief engagement with this dimension would add relevance and depth to the manuscript.*

**We sincerely thank the reviewer for this valuable suggestion. We agree that a more explicit discussion of how the findings relate to contemporary digital forms of bullying adds both relevance and interpretive depth, particularly given the central role that online spaces now play in adolescent social life. In response, we revised the Discussion section to engage more directly with the relationship between physical bullying and digital forms of peer aggression. Specifically, we now note that traditional bullying and cyberbullying often overlap rather than occurring in isolation, and we clarify that the present focus on physical bullying should not be interpreted as implying that offline aggression is disconnected from adolescents’ broader online–offline social worlds. We also added a brief future-research implication suggesting that subsequent studies examine whether the age and gender pattern observed here is reproduced across cyberbullying or hybrid forms of bullying.**

**…**

It is also worth noting that physical bullying should be interpreted in relation to contemporary digital forms of peer aggression. Prior research has shown that traditional bullying and cyberbullying often overlap rather than occurring in isolation [14,15]. Thus, although the present study focused specifically on physical bullying, the findings should not be read as implying that offline aggression is disconnected from adolescents’ broader online–offline social worlds. Instead, the persistence of gendered patterns in physical bullying suggests that embodied, face-to-face aggression remains salient even in an era when digital interaction is central to adolescent social life. Future research could examine whether the age and gender pattern observed here is reproduced across cyberbullying or hybrid forms of bullying.

*…

Discussion: Passage regarding physical skills and hormonal changes
The passage discussing adolescent boys' need for healthy environments to develop physical skills and manage competitive attitudes — and the potential consequences of inadequate guidance — raises interesting points. However, it may benefit from more explicit grounding, as aspects such as physical skill development and hormonal influences were not introduced in detail earlier in the manuscript. The authors might consider either expanding on this argument with supporting evidence in the introduction, or integrating the passage more clearly into the existing theoretical framework. As it stands, it reads somewhat as a standalone observation.*

**We sincerely thank the reviewer for this thoughtful comment. We agree that the earlier version of the passage on physical skills, competition, and hormonal or developmental change was not sufficiently well integrated into the broader theoretical framework of the manuscript and could read as a somewhat standalone observation. In response, we revised the Implications section to ground this point more carefully. Specifically, we no longer frame physical activity, puberty, or bodily development as direct causal explanations of bullying. Instead, we now clarify that the key implication concerns how school environments may either amplify or buffer the ways in which status competition and emotion regulation are enacted in peer relations. This revision better aligns the passage with the manuscript’s broader emphasis on social learning, peer dynamics, school climate, and developmental context, while avoiding stronger biological claims that cannot be directly supported by the present dataset.**

**…**

Given that the present study focuses on physical bullying, one practical implication is that schools may benefit from structured environments that channel competition, peer status, and conflict into supervised and prosocial forms of interaction. The point is not that physical activity, biological maturation, or puberty directly causes bullying, which the present study did not measure, but that school environments can either amplify or buffer how status competition and emotion regulation are enacted in peer relations. In this sense, the earlier discussion of physical skills and competitive attitudes is better understood as a contextual implication about school organization and developmental support, rather than as a direct causal claim about hormones or bodily change.

…

*Discussion: Passage regarding interventions and role models
The recommendations around healthy models of strength and the importance of teacher and parental role models — particularly in father–son and male teacher–male student relationships — are a thoughtful and meaningful way to close the discussion. To strengthen this section further, the authors might consider whether any existing programs or interventions support these recommendations, and if so, cite them. It would also be particularly compelling to connect these suggestions explicitly to the social and cultural context of the studied region — what might context-sensitive implementation look like, and what conditions would need to be in place to support it?*

**We sincerely thank the reviewer for this thoughtful and constructive suggestion. We agree that the earlier version of the manuscript did not sufficiently ground the discussion of interventions and role models in existing programmatic evidence, nor did it connect these implications clearly enough to the specific social and institutional context of the studied region. This comment was very helpful in prompting us to strengthen the closing section of the Discussion.**

**In response, we revised the Implications section in two ways. First, we now cite existing intervention literature to support the recommendation that bullying prevention should be embedded in broader school-wide efforts and, where relevant, integrated into physical education and social-emotional learning approaches. Second, we revised the discussion of role models and implementation to connect these recommendations more explicitly to the context of urban Indian schools. The revised text now emphasizes that context-sensitive implementation may require more than generic anti-bullying messaging, including consistent adult responses, emotionally safe classroom climates, and developmentally appropriate attention to peer interaction and gendered expectations. We believe these changes have made the implications section more evidence-based, contextually grounded, and responsive to the reviewer’s suggestion.**

Specific measures integrated into the physical education curriculum may be useful when they are explicitly designed for bullying prevention. However, the broader implication is not limited to physical education alone. Updated evidence from school-based intervention research suggests that anti-bullying efforts are generally more effective when they are embedded in whole-school prevention frameworks, supported by clear policies, classroom expectations, peer involvement, and parent communication [12]. Universal social-emotional learning programs may also be relevant, because they improve emotion regulation, interpersonal competence, and behavioral adjustment in ways that can support bullying prevention more broadly [64].

In the present study, male adolescents showed higher involvement in both physical bullying perpetration and physical victimization, and the Introduction framed these patterns partly through gendered socialization and social learning processes. From this perspective, school and family contexts may be important sites for reinforcing respectful forms of peer interaction, emotional regulation, and nonviolent responses to conflict. Research on school-based bullying prevention further suggests that interventions are more effective when they are integrated into broader school-wide efforts rather than limited to reactive responses to individual incidents [65,66]. Accordingly, prevention strategies may benefit from combining clear adult responses, supportive classroom climates, and opportunities for students to develop interpersonal and self-regulatory skills. In the context of the present study, this suggests that implementation in urban Indian schools may require more than generic anti-bullying messaging. It may also require school-level conditions that support consistency in adult intervention, emotionally safe classroom climates, and developmentally appropriate attention to peer interaction and gendered expectations. Because the present sample was relatively small and drawn from specific urban settings in South India, these implications should be considered preliminary rather than universally generalizable.
