# Supplementary material for: How gender is associated with physical bullying and victimization across adolescent stages
Source: PLoS One. 2026 Jun 25;21(6):e0352450. doi: 10.1371/journal.pone.0352450 (PMC13298761; doi:10.1371/journal.pone.0352450)
Supplement: Attachment — Submitted filename: 20260609 Plos ONE editor letter.docx [file pone.0352450.s003.docx]

*Plos ONE*

June 9, 2026

Dear Editors and the Editorial Office:

*Submission of a revised manuscript*

Thank you very much for spending a great amount of time and effort reviewing our manuscript. We would like to submit a revised manuscript titled “**How Gender Is Associated with Physical Bullying and Victimization Across Adolescent Stages”.**

In the revised version, we have made the following changes and articulations:

1. Methods have been enhanced to increase academic rigor and transparency.
2. Findings have been enhanced to increase readability.
3. Implications have been updated.
4. Limitations have been expanded.

In our following responses, we have integrated the issues addressed by the editor together with similar concerns raised by the reviewers. We have addressed point-to-point responses to the comments of reviewers in our updated version. Please note that since the paper has gone through a massive revision, we do not highlight the revisions in the manuscript. However, in this letter, the parts that are revised based on reviewer feedback are highlighted in yellow. Below are our modifications and answers (**emboldened**) to Reviewer 1 (*in italics*).

**First of all, we would like to express our sincere appreciation for the reviewers’ professionalism, goodwill, and meticulous review report. This is a warm feeling considering the current state of the modern academic publishing system. With their help, we have improved the manuscript significantly in many aspects. We present our responses to their detailed suggestions below.**

*Reviewer #1:*

*1. Statistical Reporting (Bayesian Uncertainty) While the inclusion of posterior density plots (Figures 2 and 4) is appreciated for visualizing parameter constraints, relying solely on graphical representations is insufficient for precise data extraction.
• Recommendation: To ensure complete statistical transparency, the authors should include numerical values for the 95% Credibility Intervals (CrI) or Highest Posterior Density Intervals (HPDI) directly within Tables 3 and 4.*

**We thank the reviewer for this helpful recommendation. We agree that posterior density plots alone are not sufficient for precise statistical interpretation. To improve statistical transparency, we have revised Tables 3 and 4 to include numerical 95% credible intervals (CrIs) for all reported posterior parameters.**

**Table 3.**

*Model 1’s simulated posteriors*

| **Parameters** | **Mean (M)** | **Standard deviation (S)** | ***95% CrI*** | ***n_eff*** | ***Rhat*** |
| --- | --- | --- | --- | --- | --- |
| Constant | 7.58 | 0.37 | (6.89, 8.30) | 8942 | 1 |
| *gender* | 0.75 | 1.09 | (-1.35, 2.82) | 6227 | 1 |
| *agecat_gender* | 0.57 | 0.55 | (-0.50, 1.63) | 6591 | 1 |

*Note. CrI = credible interval. The 95% CrI was calculated using the 2.5% and 97.5% posterior quantiles.*

**…**

**Table 4.**

*Model 2’s simulated posteriors*

| **Parameters** | **Mean (M)** | **Standard deviation (S)** | ***95% CrI*** | **n_eff** | **Rhat** |
| --- | --- | --- | --- | --- | --- |
| Constant | 8.34 | 0.49 | (7.39, 9.31) | 8844 | 1 |
| *gender* | 2.51 | 1.45 | (-0.45, 5.40) | 5980 | 1 |
| *agecat_gender* | 0.12 | 0.74 | (-1.33, 1.62) | 5929 | 1 |

*Note. CrI = credible interval. The 95% CrI was calculated using the 2.5% and 97.5% posterior quantiles.*

*2. Narrative Interpretation of Interaction Terms While the interaction terms are now mathematically specified in the equations, their practical interpretation within the text remains somewhat dense.
• Recommendation: Please add a brief, explicit narrative in the Results section that "translates" the interaction coefficients, guiding the reader on how the effect of gender fluctuates as adolescents transition across the ordinal stages of age.*

**We thank the reviewer for this important suggestion. We agree that the interaction terms required clearer substantive interpretation for readers who may not be familiar with Bayesian moderation models. We have revised the Results section to provide a more explicit narrative explanation of the interaction coefficients. Please see our revisions as below:**

**…**

The posterior mass for the gender coefficient was concentrated predominantly in the positive region, suggesting a positive tendency in the association between male gender and physical bullying, although the 95% CrI included zero. The posterior mass for the gender × age-category interaction term was also mainly positive, but more dispersed. Substantively, this suggests that the estimated male–female difference in physical bullying may become larger across later adolescent stages. In practical terms, male adolescents were estimated to report higher physical bullying than female adolescents, and this estimated gap appeared somewhat larger from early to middle and late adolescence. However, because the 95% CrI for the interaction term included zero, this pattern should be interpreted as a suggestive tendency rather than as strong evidence of a definitive moderation effect.

**…**

Figure 4 presents the joint posterior distribution of the gender coefficient and the gender × agecat coefficient. The posterior mass for the gender coefficient was concentrated predominantly in the positive region, suggesting a positive tendency in the association between male gender and physical victimization. By contrast, the posterior mass for the gender × age-category interaction term was much more diffuse and remained close to the weaker-effect region, indicating substantial uncertainty regarding age-based moderation. Substantively, this means that although male adolescents were estimated to report higher physical victimization than female adolescents, the estimated male–female difference in victimization did not clearly increase or decrease across early, middle, and late adolescence. Thus, unlike physical bullying perpetration, the gendered pattern of physical victimization appeared less sensitive to adolescent stage in this sample.

*…
3. Deepening the Discussion on Selection Bias The low parental consent return rate of 24% is a critical limitation that warrants a slightly deeper critical reflection.
• Recommendation: Please expand the Limitations section by briefly discussing the potential direction of this bias. Specifically, address whether families of highly vulnerable adolescents or those with a history of bullying might have been less likely to consent, and how this self-selection might have attenuated the observed effect sizes.*

**We thank the reviewer for encouraging a deeper treatment of the low parental consent return rate. We agree that this is an important limitation. We have expanded the Limitations section to discuss the possible direction of participation bias more explicitly.**

…

Second, among the 700 students who participated in the initial workshop, only 169, approximately 24%, submitted parental permission forms and were included in the final dataset. This low consent return rate raises an important risk of self-selection bias. It is possible that adolescents whose families were less engaged with school communication, had lower trust in institutional procedures, or were dealing with more sensitive bullying-related experiences may have been less likely to return parental consent forms. Families of highly vulnerable adolescents, or those whose children had a history of bullying perpetration or victimization, may also have been reluctant to participate because of stigma, discomfort, or fear of disclosure. If this occurred, the analytic sample may underrepresent students with more severe bullying involvement, thereby compressing the observed range of bullying and victimization scores and potentially attenuating the estimated associations. The findings should therefore be interpreted as patterns among consenting participants rather than as fully representative estimates for the broader student population.

*…
4. Final Terminological Audit Despite the excellent theoretical overhaul, a few residual phrases implying biological determinism may still linger in the text.
• Recommendation: Perform a final, rigorous proofreading of the Discussion section to ensure that terms like "natural differences" are consistently replaced with construct-appropriate language, such as "socialized behavioral patterns" or "gendered expectations".*

**We thank the reviewer for this important caution. We agree that the manuscript should avoid language that could imply biological determinism. We therefore conducted a final terminological audit of the Discussion section and revised residual phrasing that could be read as suggesting natural or biologically fixed gender differences.** **In particular, we revised the title and Discussion wording to avoid framing the findings through puberty-related or biologically deterministic language.**

*5. Data Availability Standards
• Recommendation: Ensure that the repository details or access protocols for the secondary dataset comply strictly with PLOS ONE’s open data policy, allowing independent researchers to fully replicate the Bayesian models.*

**We thank the reviewer for raising this important issue. We agree that the Data Availability Statement should provide sufficient information for independent researchers to replicate the Bayesian models. We have therefore revised the Data Availability Statement to clarify both the source of the secondary dataset and the availability of the analysis code.**

**The dataset used in this study can be accessed at** [**https://data.mendeley.com/datasets/sxf9kkb6t2/3**](https://data.mendeley.com/datasets/sxf9kkb6t2/3)**. In addition, to support reproducibility, we have uploaded the R analysis script as a supplementary file.**
