# Supplementary material for: How gender is associated with physical bullying and victimization across adolescent stages
Source: PLoS One. 2026 Jun 25;21(6):e0352450. doi: 10.1371/journal.pone.0352450 (PMC13298761; doi:10.1371/journal.pone.0352450)
Supplement: Peer review history

# Peer review: Decision Letter 0

**DOI:** 10.1371/journal.pone.0352450.r001

**Authors:** Paul Obeng

8 Apr 2026

PONE-D-25-51078

How gender affects physical bullying aggression and victimization throughout puberty in adolescents

PLOS One

Dear Dr. Jin,

Thank you for submitting your manuscript to PLOS ONE. After careful consideration, we feel that it has merit but does not fully meet PLOS ONE’s publication criteria as it currently stands. Therefore, we invite you to submit a revised version of the manuscript that addresses the points raised during the review process.

Please submit your revised manuscript by May 23 2026 11:59PM. If you will need more time than this to complete your revisions, please reply to this message or contact the journal office at plosone@plos.org. When you're ready to submit your revision, log on to https://www.editorialmanager.com/pone/ and select the 'Submissions Needing Revision' folder to locate your manuscript file.

Please include the following items when submitting your revised manuscript:

- A letter that responds to each point raised by the academic editor and reviewer(s). You should upload this letter as a separate file labeled 'Response to Reviewers'.
- A marked-up copy of your manuscript that highlights changes made to the original version. You should upload this as a separate file labeled 'Revised Manuscript with Track Changes'.
- An unmarked version of your revised paper without tracked changes. You should upload this as a separate file labeled 'Manuscript'.

If you would like to make changes to your financial disclosure, please include your updated statement in your cover letter. Guidelines for resubmitting your figure files are available below the reviewer comments at the end of this letter.

If applicable, we recommend that you deposit your laboratory protocols in protocols.io to enhance the reproducibility of your results. Protocols.io assigns your protocol its own identifier (DOI) so that it can be cited independently in the future. For instructions see: https://journals.plos.org/plosone/s/submission-guidelines#loc-laboratory-protocols. Additionally, PLOS ONE offers an option for publishing peer-reviewed Lab Protocol articles, which describe protocols hosted on protocols.io. Read more information on sharing protocols at https://plos.org/protocols?utm_medium=editorial-email&utm_source=authorletters&utm_campaign=protocols.

As the corresponding author, your ORCID iD is verified in the submission system and will appear in the published article. PLOS supports the use of ORCID, and we encourage all coauthors to register for an ORCID iD and use it as well. Please encourage your coauthors to verify their ORCID iD within the submission system before final acceptance, as unverified ORCID iDs will not appear in the published article. *Only*  the individual author can complete the verification step; PLOS staff *cannot*  verify ORCID iDs on behalf of authors.

We look forward to receiving your revised manuscript.

Kind regards,

Paul Obeng, MEd, MSc., M.Phil.

Academic Editor

PLOS One

Journal Requirements:

When submitting your revision, we need you to address these additional requirements.

1.Please ensure that your manuscript meets PLOS ONE's style requirements, including those for file naming. The PLOS ONE style templates can be found at

https://journals.plos.org/plosone/s/file?id=wjVg/PLOSOne_formatting_sample_main_body.pdf and

https://journals.plos.org/plosone/s/file?id=ba62/PLOSOne_formatting_sample_title_authors_affiliations.pdf

2. Thank you for uploading your study's underlying data set. Unfortunately, the repository you have noted in your Data Availability statement does not qualify as an acceptable data repository according to PLOS's standards.

At this time, please upload the minimal data set necessary to replicate your study's findings to a stable, public repository (such as figshare or Dryad) and provide us with the relevant URLs, DOIs, or accession numbers that may be used to access these data. For a list of recommended repositories and additional information on PLOS standards for data deposition, please see https://journals.plos.org/plosone/s/recommended-repositories.

3. Please note that your Data Availability Statement is currently missing the repository name and/or the DOI/accession number of each dataset OR a direct link to access each database. If your manuscript is accepted for publication, you will be asked to provide these details on a very short timeline. We therefore suggest that you provide this information now, though we will not hold up the peer review process if you are unable.

4. Your ethics statement should only appear in the Methods section of your manuscript. If your ethics statement is written in any section besides the Methods, please move it to the Methods section and delete it from any other section. Please ensure that your ethics statement is included in your manuscript, as the ethics statement entered into the online submission form will not be published alongside your manuscript.

5. We notice that your supplementary figures are uploaded with the file type 'Figure'. Please amend the file type to 'Supporting Information'. Please ensure that each Supporting Information file has a legend listed in the manuscript after the references list.

6. If the reviewer comments include a recommendation to cite specific previously published works, please review and evaluate these publications to determine whether they are relevant and should be cited. There is no requirement to cite these works unless the editor has indicated otherwise.

[Note: HTML markup is below. Please do not edit.]

Reviewer's Responses to Questions

**Comments to the Author**

1. Is the manuscript technically sound, and do the data support the conclusions?

The manuscript must describe a technically sound piece of scientific research with data that supports the conclusions. Experiments must have been conducted rigorously, with appropriate controls, replication, and sample sizes. The conclusions must be drawn appropriately based on the data presented.

Reviewer #1: Partly

Reviewer #2: Yes

**********

2. Has the statistical analysis been performed appropriately and rigorously?

Reviewer #1: I Don't Know

Reviewer #2: I Don't Know

**********

3. Have the authors made all data underlying the findings in their manuscript fully available?

The PLOS Data policy requires authors to make all data underlying the findings described in their manuscript fully available without restriction, with rare exception (please refer to the Data Availability Statement in the manuscript PDF file). The data should be provided as part of the manuscript or its supporting information, or deposited to a public repository. For example, in addition to summary statistics, the data points behind means, medians and variance measures should be available. If there are restrictions on publicly sharing data—e.g. participant privacy or use of data from a third party—those must be specified.

Reviewer #1: Yes

Reviewer #2: Yes

**********

4. Is the manuscript presented in an intelligible fashion and written in standard English?

PLOS ONE does not copyedit accepted manuscripts, so the language in submitted articles must be clear, correct, and unambiguous. Any typographical or grammatical errors should be corrected at revision, so please note any specific errors here.

Reviewer #1: Yes

Reviewer #2: Yes

**********

5. Review Comments to the Author

Please use the space provided to explain your answers to the questions above. You may also include additional comments for the author, including concerns about dual publication, research ethics, or publication ethics. (Please upload your review as an attachment if it exceeds 20,000 characters)

Reviewer #1: 1. Summary

This manuscript examines gender differences in physical bullying perpetration and victimization among adolescents using secondary data from 169 students in India. The authors apply Bayesian regression models using Markov Chain Monte Carlo (MCMC) simulations to test whether gender predicts physical bullying behaviors and whether age moderates these relationships.

The results suggest that male adolescents show higher levels of both physical bullying aggression and victimization compared to females. Age appears to moderate the relationship between gender and bullying aggression but not victimization.

The topic is relevant and socially important, and the manuscript demonstrates an effort toward transparent statistical reporting. However, several methodological and reporting issues should be addressed before the manuscript is suitable for publication.

From a feminist perspective, the manuscript could benefit from a more critical engagement with gender as a social construct rather than treating it primarily as a binary demographic variable. The analysis relies on a male/female categorization and interprets differences largely through behavioral tendencies, which risks reinforcing essentialist interpretations of gendered aggression. Feminist scholarship has extensively shown that bullying behaviors are embedded within broader systems of gendered power relations, hegemonic masculinity, and patriarchal social norms rather than merely reflecting individual characteristics. While the authors briefly reference masculinity norms, the analysis could be strengthened by situating physical bullying within these structural dynamics, including how school environments reproduce gender hierarchies and normalize certain forms of male aggression. Additionally, the absence of participants identifying outside the gender binary is treated as a methodological limitation but not explored analytically; feminist and queer research suggests that gender-diverse students often experience disproportionate bullying. Incorporating this perspective would deepen the theoretical framework and prevent interpretations that inadvertently naturalize gender differences in aggression.

2. Major Comments

• Limited novelty relative to existing literature: The main finding—that males show higher levels of physical bullying and victimization—has been widely documented in previous studies. The authors should better clarify the specific contribution of this study and how it advances knowledge beyond existing findings.

• Small and potentially biased sample: The analysis is based on 169 participants from two cities in India. Only about 24% of the initial workshop participants submitted parental consent forms, which may introduce participation bias. The authors should discuss how this affects the generalizability of the results.

• Model specification is unclear: The regression equations presented contain notation inconsistencies and potential specification problems. The interaction term between age and gender should be clearly defined, and the authors should clarify whether age was treated as categorical or ordinal.

• Weak interpretation of Bayesian results: The manuscript reports posterior means and standard deviations but does not include credible intervals or posterior probabilities. These are essential elements of Bayesian reporting and should be included.

• Overinterpretation of cultural explanations: Some explanations related to gender norms and Hindu traditions are speculative because these variables were not measured in the dataset. Such interpretations should be presented more cautiously.

• Justification for Bayesian methods: The authors state that Bayesian analysis was chosen due to the small sample size, but the methodological rationale should be expanded. It would be helpful to explain why Bayesian modeling is preferable in this context.

3. Minor Comments

• Language editing: The manuscript would benefit from professional English editing to improve clarity and grammar.

• Abstract: The abstract should explicitly mention the study design (secondary data analysis), sample size (N = 169), and the use of Bayesian regression models.

• Figures: Several diagnostic plots are included in the manuscript. Some of these could be moved to supplementary material to improve readability.

• Terminology: The terms 'physical bullying aggression' and 'physical bullying' are used inconsistently and should be standardized.

• Ethics statement: Since the study relies on secondary data, the authors should clarify whether additional ethical approval was required and confirm that the dataset was fully anonymized.

4. Recommendation

Decision: Major Revision.

The manuscript addresses an important topic and applies transparent analytical procedures. However, significant revisions are required regarding model specification, statistical reporting, and interpretation of results before the manuscript can be considered for publication.

Reviewer #2: Strengths

This manuscript addresses an important and timely topic, and the authors are to be commended for conducting their study in a non-WEIRD region, making a valuable contribution to a more globally diverse research landscape. The inclusion of gender and age differences as well as their interactions is a particular strength, as it opens up meaningful possibilities for the development of targeted intervention strategies. The study's commitment to open science principles in terms of data and analysis transparency is also very much appreciated.

//

Suggestions for revision

General: Socioeconomic status (SES)

Since SES was collected as a variable, readers may wonder about its potential role in the observed patterns of bullying aggression and victimization. It would be helpful if the authors could either incorporate SES more explicitly into the analysis and discussion, or briefly explain their reasoning for not doing so.

Introduction: Regional and contextual specificity

The manuscript would benefit from a richer engagement with the specific cultural, social, and institutional context of the study setting. This might include regional particularities in school norms, local social norms around bullying, and potentially the influence of religion on social behavior. Some of this context does appear in the discussion, which is great — the authors might consider introducing elements of it already in the introduction to help orient the reader from the outset.

Introduction: Theoretical depth on gender norms

The section on gender norms has good foundations, but could be enriched by engaging more deeply with relevant theoretical frameworks. At present, the discussion focuses primarily on norms around normatively "masculine" behavior, and there is room to broaden this. For example, drawing on Bandura's social learning theory, the role of behavioral modeling by (male) teachers could be explored more explicitly. It would also be valuable to consider the developmental-psychological and physiological challenges that adolescent boys navigate during puberty, and how these may shape their social roles within the school context in ways that could contribute to bullying dynamics. Framing the phenomenon within a biopsychosocial model could provide a useful integrative structure for these considerations.

Introduction/Methods: Positionality and contextualization

A brief reflexive statement on the positionality of the research team would be a welcome addition. It would help readers understand the perspective from which masculinity and gender norms are conceptualized, and how the authors' own standpoints may have informed the framing of the research questions and the interpretation of findings.

Discussion: Digital forms of bullying

It would be worth reflecting more explicitly in the discussion on how the findings relate to contemporary digital forms of bullying. Given how central online spaces have become to adolescent social life, even a brief engagement with this dimension would add relevance and depth to the manuscript.

Discussion: Passage regarding physical skills and hormonal changes

The passage discussing adolescent boys' need for healthy environments to develop physical skills and manage competitive attitudes — and the potential consequences of inadequate guidance — raises interesting points. However, it may benefit from more explicit grounding, as aspects such as physical skill development and hormonal influences were not introduced in detail earlier in the manuscript. The authors might consider either expanding on this argument with supporting evidence in the introduction, or integrating the passage more clearly into the existing theoretical framework. As it stands, it reads somewhat as a standalone observation.

Discussion: Passage regarding interventions and role models

The recommendations around healthy models of strength and the importance of teacher and parental role models — particularly in father–son and male teacher–male student relationships — are a thoughtful and meaningful way to close the discussion. To strengthen this section further, the authors might consider whether any existing programs or interventions support these recommendations, and if so, cite them. It would also be particularly compelling to connect these suggestions explicitly to the social and cultural context of the studied region — what might context-sensitive implementation look like, and what conditions would need to be in place to support it?

**********

6. PLOS authors have the option to publish the peer review history of their article (what does this mean?). If published, this will include your full peer review and any attached files.

If you choose “no”, your identity will remain anonymous but your review may still be made public.

**Do you want your identity to be public for this peer review?**  For information about this choice, including consent withdrawal, please see our Privacy Policy.

Reviewer #1: No

Reviewer #2: No

**********

[NOTE: If reviewer comments were submitted as an attachment file, they will be attached to this email and accessible via the submission site. Please log into your account, locate the manuscript record, and check for the action link "View Attachments". If this link does not appear, there are no attachment files.]

To ensure your figures meet our technical requirements, please review our figure guidelines: https://journals.plos.org/plosone/s/figures

You may also use PLOS’s free figure tool, NAAS, to help you prepare publication quality figures: https://journals.plos.org/plosone/s/figures#loc-tools-for-figure-preparation.

NAAS will assess whether your figures meet our technical requirements by comparing each figure against our figure specifications.

# Author comment: Author response to Decision Letter 1

**DOI:** 10.1371/journal.pone.0352450.r002

15 Apr 2026

Plos ONE

April 15, 2026

Dear Editors and the Editorial Office:

Submission of a revised manuscript

Thank you very much for spending a great amount of time and effort reviewing our manuscript. We would like to submit a revised manuscript titled “How gender is associated with physical bullying and victimization throughout puberty in adolescents”.

In the revised version, we have made the following changes and articulations:

Introduction has been rewritten.

Methods have been enhanced to increase academic rigor and transparency.

Findings have been reorganized and enhanced.

Discussions have been streamlined and improved.

Implications have been updated.

Limitations have been expanded.

In our following responses, we have integrated the issues addressed by the editor together with similar concerns raised by the reviewers. We have addressed point-to-point responses to the comments of reviewers in our updated version. Please note that since the paper has gone through a massive revision, we do not highlight the revisions in the manuscript. However, in this letter, the parts that are revised based on reviewer feedback are highlighted in yellow. Below are our modifications and answers (emboldened) to Reviewer 1 and Reviewer 2 (in italics).

First of all, we would like to express our sincere appreciation for the reviewers’ professionalism, goodwill, and meticulous review report. This is a warm feeling considering the current state of the modern academic publishing system. With their help, we have improved the manuscript significantly in many aspects. We present our responses to their detailed suggestions below.

Reviewer #1:

1. Summary

This manuscript examines gender differences in physical bullying perpetration and victimization among adolescents using secondary data from 169 students in India. The authors apply Bayesian regression models using Markov Chain Monte Carlo (MCMC) simulations to test whether gender predicts physical bullying behaviors and whether age moderates these relationships.

The results suggest that male adolescents show higher levels of both physical bullying aggression and victimization compared to females. Age appears to moderate the relationship between gender and bullying aggression but not victimization.

The topic is relevant and socially important, and the manuscript demonstrates an effort toward transparent statistical reporting. However, several methodological and reporting issues should be addressed before the manuscript is suitable for publication.

We sincerely thank you for the kind words and encouragement.

From a feminist perspective, the manuscript could benefit from a more critical engagement with gender as a social construct rather than treating it primarily as a binary demographic variable. The analysis relies on a male/female categorization and interprets differences largely through behavioral tendencies, which risks reinforcing essentialist interpretations of gendered aggression. Feminist scholarship has extensively shown that bullying behaviors are embedded within broader systems of gendered power relations, hegemonic masculinity, and patriarchal social norms rather than merely reflecting individual characteristics. While the authors briefly reference masculinity norms, the analysis could be strengthened by situating physical bullying within these structural dynamics, including how school environments reproduce gender hierarchies and normalize certain forms of male aggression. Additionally, the absence of participants identifying outside the gender binary is treated as a methodological limitation but not explored analytically; feminist and queer research suggests that gender-diverse students often experience disproportionate bullying. Incorporating this perspective would deepen the theoretical framework and prevent interpretations that inadvertently naturalize gender differences in aggression.

We sincerely thank the reviewer for this thoughtful and important comment. We greatly appreciate the feminist and queer perspective brought to the manuscript, as it helped us recognize that the earlier version did not engage sufficiently with gender as a socially constructed and institutionally mediated phenomenon. This comment substantially improved the depth, theoretical sensitivity, and overall interpretive quality of the paper.

In response, we have revised the manuscript in several ways. First, we reworked the Introduction to move beyond treating gender primarily as a binary demographic variable and instead situate physical bullying within broader systems of gender regulation, hegemonic masculinity, and school-based socialization. In doing so, we incorporated feminist and queer scholarship to emphasize that bullying may reflect not only individual behavior but also gendered power relations reproduced through peer culture and school environments. Second, we revised the theoretical framing to clarify that observed male–female differences should not be interpreted as naturalized or essential behavioral tendencies. Third, we expanded our treatment of the binary structure of the dataset. Rather than presenting the absence of participants outside the gender binary only as a methodological limitation, we now acknowledge its analytic significance and note that gender-diverse adolescents often experience disproportionately high levels of bullying and victimization, but fall outside the scope of the present data. Finally, we revised the Discussion to avoid overinterpreting gender differences as direct reflections of fixed behavioral traits and instead interpret them more cautiously in relation to school context, peer dynamics, and wider sociocultural norms. Please see our revisions in the Introduction, Discussion, and Limitations

Introduction

Bullying is a pervasive issue among adolescents, with far-reaching consequences for both victims and perpetrators [1,2]. Bullying is a repeated aggressive behavior involving an interpersonal imbalance of power, where the perpetrator intentionally inflicts harm, either physically, verbally, or psychologically, on the victim [3–5]. The main types of bullying include physical (e.g. hitting, kicking, pushing), verbal (e.g. name-calling, taunting, threatening), relational (e.g. spreading rumors, social exclusion), and cyberbullying (e.g. using electronic means to harass or intimidate) [6]. Bullying behaviors are relatively prevalent in adolescents and can have severe psychological, physical, and emotional consequences for victims such as depression, anxiety, low self-esteem, fear, and absenteeism from school [7].

Physical bullying is a form of direct aggression that involves the use of physical force or intimidation to harm others [8]. Cross-national research has documented substantial between-country variation in bullying, with relatively high prevalence reported in many lower- and middle-income settings, although the pattern differs by bullying form, country context, and measurement approach [9–11]. One possible explanation is that some education systems have more developed school-wide prevention infrastructures, stronger staff training, and clearer anti-bullying policy frameworks, whereas implementation capacity and evaluated intervention evidence remain more limited in many low- and middle-income settings [12,13]. Although cyberbullying has become a major concern in recent years, physical bullying remains highly consequential for adolescent well-being. [14] found that many adolescents who experience cyberbullying also experience traditional forms of bullying, while [15] likewise showed that online and offline victimization often overlap. Physical bullying therefore remains an important and distinct dimension of adolescent peer relations rather than a phenomenon displaced by digital forms of aggression.

Regarding gender, prior research has consistently shown that boys are more likely than girls to be involved in physical forms of bullying, both as perpetrators and as victims [4,16]. Similar patterns have been reported across diverse national contexts, including the United States [17], China [18], Brazil [19], and Saudi Arabia [20]. However, gender should not be understood merely as a binary demographic characteristic that mechanically predicts aggression. Rather, feminist and queer scholarship suggests that bullying is often embedded in wider systems of gender regulation through which peer cultures and school institutions reproduce hierarchies of masculinity, femininity, and heterosexuality [21–23]. From a theoretical perspective, social learning theory remains relevant because it helps explain how students learn not only from direct experience but also from observing which behaviors are modeled, tolerated, and rewarded in their environments [24]. In this sense, teachers, alongside peers, form part of the social environment through which gendered conduct is reinforced or challenged; school-based research on adolescent masculinities has similarly emphasized the importance of positive role models and developmental programming in shaping boys’ understandings of masculinity [25], even as scholarship on male teachers cautions that such role-model effects are socially mediated rather than automatic [26]. As a result, in many school and peer contexts, boys may face stronger pressure to display toughness, dominance, and emotional restraint, whereas students who depart from normative gender expectations may become especially exposed to ridicule, harassment, or exclusion [27,28]. From this perspective, physical bullying can function not only as individual aggression but also as a socially meaningful practice through which toughness, dominance, and normative masculinity are enacted, recognized, and sometimes rewarded in school settings [29].

Prior studies on the relationship between bullying and age, however, have produced less consistent findings. Some studies suggest that bullying involvement increases with age, especially when externalizing behaviors such as aggression and defiance become more common in later stages of childhood and adolescence [30]. Other research has reported a downward trend, arguing that bullying may rise during school transitions but decline as adolescents mature cognitively and as peer norms shift [31,32]. [15] further showed that age-related patterns may differ across bullying types and across traditional versus online contexts. These mixed findings suggest that age may not operate as a simple predictor. Rather, adolescence is a developmental period marked by rapid bodily change, increased sensitivity to peer evaluation, status concerns, and evolving social identities[33–35], all of which may shape how gendered patterns of physical bullying emerge and are expressed.

Despite the substantial literature on bullying, the present study addresses a more specific question. Much of the existing literature has examined bullying in broad terms or has focused primarily on overall prevalence and correlates, whereas physical bullying may operate through somewhat distinct social dynamics because it is more visibly tied to bodily dominance, vulnerability, and peer-status displays [8,29]. In addition, although gender and age have both been widely studied, it remains less clear whether the commonly observed gender pattern in physical bullying and victimization varies across developmental stages of adolescence.

This question is especially relevant in the Indian context, where bullying research has expanded but remains marked by important contextual diversity[36,37]. A systematic review of bullying and victimization among adolescents in India suggests that bullying in Indian schools is shaped by multiple school, peer, and family factors rather than by individual traits alone [37]. A more recent scoping review similarly argues that bullying patterns in India are influenced by factors aligned with the broader cultural climate, underscoring the need to situate bullying within wider sociocultural contexts [36]. Therefore, the present study not only provides evidence from a non-WEIRD context, but also situates the analysis in a setting where rapid urbanization, educational change, and digitalization coexist with persistent yet evolving gender norms [38,39]. This is particularly relevant for urban school settings, because Indian schools are also sites where gender norms are reproduced, negotiated, and sometimes reshaped [40,41]. In addition, recent evidence from southern India highlights the relevance of region, school setting, and classroom context in bullying-related experiences, suggesting that urban South Indian school environments deserve closer attention rather than being treated as interchangeable with other settings [42]. Against this background, data from school-going adolescents in Chennai and Bengaluru are useful not because prior work has already fully mapped those cities’ bullying subcultures, but because they provide an opportunity to examine whether established gender patterns in physical bullying appear similarly in contemporary urban South India.

The present study contributes to the literature in three ways. First, it focuses specifically on physical bullying, rather than treating bullying as a single undifferentiated construct. Second, it examines not only gender differences but also whether age moderates the association between gender and both physical bullying and physical victimization. Third, it uses recent secondary data from school-going adolescents in Chennai and Bengaluru, thereby extending discussion beyond the predominantly Western literature and reassessing established patterns in a non-WEIRD context. Because the available dataset records gender only in male/female categories, the present study examines gendered differences as captured within a binary dataset. Thus, using secondary survey data collected in 2022 from school-going adolescents in Chennai and Bengaluru, India, the present study addresses the following research objectives:

1. To examine the association between gender and physical bullying among adolescents and to assess whether age moderates this relationship.

2. To investigate the association between gender and physical bullying victimization among adolescents and to determine whether age moderates this relationship.

Discussion

The analysis results show that male adolescents are more likely to report physical bullying and physical victimization than female adolescents. In addition, the results suggest that the positive association between male gender and physical bullying becomes more pronounced across later adolescent stages in this sample, whereas the association between gender and physical victimization does not show a comparably clear age-based moderation pattern. Because the present study is based on secondary cross-sectional data, these findings should be interpreted as associations rather than as direct evidence of causal developmental mechanisms.

4.1 Discussion of RQ1

In the data article from which the present study retrieved the dataset [43], the authors presented a brief table of multiple linear regression across variables, but did not include the gender factor and did not find any significant correlation between age and physical bullying as well as physical victimization. The present study explored the influence of gender and the moderating effect of age on such aspects of bullying experiences. Our results on the gender differences in physical bullying and victimization are in alignment with prior findings [4,16,17,56]. One possible explanation is that gendered socialization processes, where society expects boys to be tough, dominant, and physically assertive, while girls are often encouraged to be nurturing and conflict‐avoidant, play a crucial role in shaping bullying behaviors [28,29]. For instance, research has shown that traditional masculinity norms are linked to higher physical aggression in boys because they valorize strength and assertiveness [29].

The age moderation observed in Model 1 suggests that the association between male gender and physical bullying may become more pronounced across later adolescent stages in this sample. One plausible interpretation is that, as adolescents grow older, peer status concerns, susceptibility to peer influence, and school-based expectations around masculinity may become more

Attachment Submitted filename: 20260415 reviewer 2.docx

# Peer review: Decision Letter 1

**DOI:** 10.1371/journal.pone.0352450.r003

**Authors:** Paul Obeng, Javier Fagundo-Rivera

8 Jun 2026

PONE-D-25-51078R1

How gender is associated with physical bullying and victimization throughout puberty in adolescents

PLOS One

Dear Dr. Jin,

Thank you for submitting your manuscript to PLOS ONE. After careful consideration, we feel that it has merit but does not fully meet PLOS ONE’s publication criteria as it currently stands. Therefore, we invite you to submit a revised version of the manuscript that addresses the points raised during the review process.

==============================

**ACADEMIC EDITOR:**

Dear Authors,

Thank you for submitting your manuscript entitled “ How gender is associated with physical bullying and victimization throughout puberty in adolescents ” (Manuscript Number: PONE-D-25-51078R1) to Plos ONE.

After evaluation and editorial assessment, the current decision is Minor Revision.

We recognize the relevance, originality, and potential contribution of this work. Overall, the manuscript is well prepared and addresses an important topic. However, several points were identified that would benefit from clarification and minor improvement before the manuscript can be considered for publication.

Please revise the manuscript carefully by addressing each comment point-by-point in a detailed response letter.

We encourage you to clearly indicate all modifications made in the revised manuscript, preferably using track changes or highlighted text.

We look forward to receiving your revised submission and appreciate your contribution to the field.

Sincerely,

Javier Fagundo-Rivera, PhD

Academic Editor

==============================

Please submit your revised manuscript by Jul 23 2026 11:59PM. If you will need more time than this to complete your revisions, please reply to this message or contact the journal office at plosone@plos.org. When you're ready to submit your revision, log on to https://www.editorialmanager.com/pone/ and select the 'Submissions Needing Revision' folder to locate your manuscript file.

Please include the following items when submitting your revised manuscript:

- A letter that responds to each point raised by the academic editor and reviewer(s). You should upload this letter as a separate file labeled 'Response to Reviewers'.
- A marked-up copy of your manuscript that highlights changes made to the original version. You should upload this as a separate file labeled 'Revised Manuscript with Track Changes'.
- An unmarked version of your revised paper without tracked changes. You should upload this as a separate file labeled 'Manuscript'.

If you would like to make changes to your financial disclosure, please include your updated statement in your cover letter. Guidelines for resubmitting your figure files are available below the reviewer comments at the end of this letter.

If applicable, we recommend that you deposit your laboratory protocols in protocols.io to enhance the reproducibility of your results. Protocols.io assigns your protocol its own identifier (DOI) so that it can be cited independently in the future. For instructions see: https://journals.plos.org/plosone/s/submission-guidelines#loc-laboratory-protocols. Additionally, PLOS ONE offers an option for publishing peer-reviewed Lab Protocol articles, which describe protocols hosted on protocols.io. Read more information on sharing protocols at https://plos.org/protocols?utm_medium=editorial-email&utm_source=authorletters&utm_campaign=protocols.

As the corresponding author, your ORCID iD is verified in the submission system and will appear in the published article. PLOS supports the use of ORCID, and we encourage all coauthors to register for an ORCID iD and use it as well. Please encourage your coauthors to verify their ORCID iD within the submission system before final acceptance, as unverified ORCID iDs will not appear in the published article. *Only*  the individual author can complete the verification step; PLOS staff *cannot*  verify ORCID iDs on behalf of authors.

We look forward to receiving your revised manuscript.

Kind regards,

Javier Fagundo-Rivera, PhD

Academic Editor

PLOS One

**Journal Requirements:**

1. If the reviewer comments include a recommendation to cite specific previously published works, please review and evaluate these publications to determine whether they are relevant and should be cited. There is no requirement to cite these works unless the editor has indicated otherwise.

2. Please review your reference list to ensure that it is complete and correct. If you have cited papers that have been retracted, please include the rationale for doing so in the manuscript text, or remove these references and replace them with relevant current references. Any changes to the reference list should be mentioned in the rebuttal letter that accompanies your revised manuscript. If you need to cite a retracted article, indicate the article’s retracted status in the References list and also include a citation and full reference for the retraction notice.

**Additional Editor Comments:**

Dear Authors,

Thank you for submitting your manuscript entitled “ How gender is associated with physical bullying and victimization throughout puberty in adolescents ” (Manuscript Number: PONE-D-25-51078R1) to Plos ONE.

After evaluation and editorial assessment, the current decision is Minor Revision.

We recognize the relevance, originality, and potential contribution of this work. Overall, the manuscript is well prepared and addresses an important topic. However, several points were identified that would benefit from clarification and minor improvement before the manuscript can be considered for publication.

Please revise the manuscript carefully by addressing each comment point-by-point in a detailed response letter.

We encourage you to clearly indicate all modifications made in the revised manuscript, preferably using track changes or highlighted text.

We look forward to receiving your revised submission and appreciate your contribution to the field.

Sincerely,

Javier Fagundo-Rivera, PhD

Academic Editor

[Note: HTML markup is below. Please do not edit.]

Reviewers' comments:

Reviewer's Responses to Questions

**Comments to the Author**

1. If the authors have adequately addressed your comments raised in a previous round of review and you feel that this manuscript is now acceptable for publication, you may indicate that here to bypass the “Comments to the Author” section, enter your conflict of interest statement in the “Confidential to Editor” section, and submit your "Accept" recommendation.

Reviewer #1: All comments have been addressed

**********

2. Is the manuscript technically sound, and do the data support the conclusions?

The manuscript must describe a technically sound piece of scientific research with data that supports the conclusions. Experiments must have been conducted rigorously, with appropriate controls, replication, and sample sizes. The conclusions must be drawn appropriately based on the data presented.

Reviewer #1: Yes

**********

3. Has the statistical analysis been performed appropriately and rigorously?

Reviewer #1: Yes

**********

4. Have the authors made all data underlying the findings in their manuscript fully available?

The PLOS Data policy requires authors to make all data underlying the findings described in their manuscript fully available without restriction, with rare exception (please refer to the Data Availability Statement in the manuscript PDF file). The data should be provided as part of the manuscript or its supporting information, or deposited to a public repository. For example, in addition to summary statistics, the data points behind means, medians and variance measures should be available. If there are restrictions on publicly sharing data—e.g. participant privacy or use of data from a third party—those must be specified.

Reviewer #1: Yes

**********

5. Is the manuscript presented in an intelligible fashion and written in standard English?

PLOS ONE does not copyedit accepted manuscripts, so the language in submitted articles must be clear, correct, and unambiguous. Any typographical or grammatical errors should be corrected at revision, so please note any specific errors here.

Reviewer #1: Yes

**********

6. Review Comments to the Author

Please use the space provided to explain your answers to the questions above. You may also include additional comments for the author, including concerns about dual publication, research ethics, or publication ethics. (Please upload your review as an attachment if it exceeds 20,000 characters)

**Reviewer #1:**

Manuscript Number: PONE-D-25-51078

Title: How gender is associated with physical bullying and victimization throughout puberty in adolescents

Journal: PLOS ONE

Recommendation: Minor Revision

General Comments

The authors have undertaken a commendable effort in revising this manuscript (R1). The incorporation of a structural framework that addresses the complexities of physical bullying in a contemporary, non-WEIRD urban Indian setting is highly valuable. The shift away from biological essentialism toward a nuanced gender-socialization perspective significantly elevates the theoretical merit of the paper. Additionally, the statistical clarification regarding the ordinal nature of the age variable and the explicit mathematical definition of the interaction terms have greatly improved the manuscript's methodological transparency.

While the majority of the previous concerns have been successfully addressed, a few minor technical and reporting adjustments are still required to meet the journal's rigorous reporting standards before final publication.

Major Achievements in the Current Revision (R1)

• Theoretical Reframing: The integration of a feminist and sociological perspective successfully contextualizes physical bullying within the dynamics of hegemonic masculinity and gendered expectations, rather than treating gender as a static biological binary.

• Methodological Clarification: Specifying age as an ordinal variable across three distinct developmental stages provides the necessary precision for the Bayesian regression models.

• Contextualization and Scope: The manuscript now clearly articulates its unique empirical contribution (focusing strictly on physical bullying in an understudied geographic region) and has appropriately removed unmeasured cultural generalizations.

• Transparency of Limitations: The authors openly acknowledge the constraints of the secondary dataset regarding non-binary gender identities and transparently report the low parental consent rate (24%).

Minor Points and Aspects Still to be Improved

1. Statistical Reporting (Bayesian Uncertainty) While the inclusion of posterior density plots (Figures 2 and 4) is appreciated for visualizing parameter constraints, relying solely on graphical representations is insufficient for precise data extraction.

• Recommendation: To ensure complete statistical transparency, the authors should include numerical values for the 95% Credibility Intervals (CrI) or Highest Posterior Density Intervals (HPDI) directly within Tables 3 and 4.

2. Narrative Interpretation of Interaction Terms While the interaction terms are now mathematically specified in the equations, their practical interpretation within the text remains somewhat dense.

• Recommendation: Please add a brief, explicit narrative in the Results section that "translates" the interaction coefficients, guiding the reader on how the effect of gender fluctuates as adolescents transition across the ordinal stages of age.

3. Deepening the Discussion on Selection Bias The low parental consent return rate of 24% is a critical limitation that warrants a slightly deeper critical reflection.

• Recommendation: Please expand the Limitations section by briefly discussing the potential direction of this bias. Specifically, address whether families of highly vulnerable adolescents or those with a history of bullying might have been less likely to consent, and how this self-selection might have attenuated the observed effect sizes.

4. Final Terminological Audit Despite the excellent theoretical overhaul, a few residual phrases implying biological determinism may still linger in the text.

• Recommendation: Perform a final, rigorous proofreading of the Discussion section to ensure that terms like "natural differences" are consistently replaced with construct-appropriate language, such as "socialized behavioral patterns" or "gendered expectations".

5. Data Availability Standards

• Recommendation: Ensure that the repository details or access protocols for the secondary dataset comply strictly with PLOS ONE’s open data policy, allowing independent researchers to fully replicate the Bayesian models.

Conclusion:

This is a high-quality manuscript that has been thoroughly improved. Addressing these final minor adjustments will ensure the paper achieves maximum clarity, transparency, and impact upon publication.

**********

7. PLOS authors have the option to publish the peer review history of their article (what does this mean?). If published, this will include your full peer review and any attached files.

If you choose “no”, your identity will remain anonymous but your review may still be made public.

**Do you want your identity to be public for this peer review?**  For information about this choice, including consent withdrawal, please see our Privacy Policy.

Reviewer #1: No

**********

[NOTE: If reviewer comments were submitted as an attachment file, they will be attached to this email and accessible via the submission site. Please log into your account, locate the manuscript record, and check for the action link "View Attachments". If this link does not appear, there are no attachment files.]

To ensure your figures meet our technical requirements, please review our figure guidelines: https://journals.plos.org/plosone/s/figures

You may also use PLOS’s free figure tool, NAAS, to help you prepare publication quality figures: https://journals.plos.org/plosone/s/figures#loc-tools-for-figure-preparation.

NAAS will assess whether your figures meet our technical requirements by comparing each figure against our figure specifications.

# Author comment: Author response to Decision Letter 2

**DOI:** 10.1371/journal.pone.0352450.r004

9 Jun 2026

Plos ONE

June 9, 2026

Dear Editors and the Editorial Office:

Submission of a revised manuscript

Thank you very much for spending a great amount of time and effort reviewing our manuscript. We would like to submit a revised manuscript titled “How Gender Is Associated with Physical Bullying and Victimization Across Adolescent Stages”.

In the revised version, we have made the following changes and articulations:

1. Methods have been enhanced to increase academic rigor and transparency.

2. Findings have been enhanced to increase readability.

3. Implications have been updated.

4. Limitations have been expanded.

In our following responses, we have integrated the issues addressed by the editor together with similar concerns raised by the reviewers. We have addressed point-to-point responses to the comments of reviewers in our updated version. Please note that since the paper has gone through a massive revision, we do not highlight the revisions in the manuscript. However, in this letter, the parts that are revised based on reviewer feedback are highlighted in yellow. Below are our modifications and answers (emboldened) to Reviewer 1 (in italics).

First of all, we would like to express our sincere appreciation for the reviewers’ professionalism, goodwill, and meticulous review report. This is a warm feeling considering the current state of the modern academic publishing system. With their help, we have improved the manuscript significantly in many aspects. We present our responses to their detailed suggestions below.

Reviewer #1:

1. Statistical Reporting (Bayesian Uncertainty) While the inclusion of posterior density plots (Figures 2 and 4) is appreciated for visualizing parameter constraints, relying solely on graphical representations is insufficient for precise data extraction.

• Recommendation: To ensure complete statistical transparency, the authors should include numerical values for the 95% Credibility Intervals (CrI) or Highest Posterior Density Intervals (HPDI) directly within Tables 3 and 4.

We thank the reviewer for this helpful recommendation. We agree that posterior density plots alone are not sufficient for precise statistical interpretation. To improve statistical transparency, we have revised Tables 3 and 4 to include numerical 95% credible intervals (CrIs) for all reported posterior parameters.

Table 3.

Model 1’s simulated posteriors

Parameters Mean (M) Standard deviation (S) 95% CrI n_eff Rhat

Constant 7.58 0.37 (6.89, 8.30) 8942 1

gender 0.75 1.09 (-1.35, 2.82) 6227 1

agecat_gender 0.57 0.55 (-0.50, 1.63) 6591 1

Note. CrI = credible interval. The 95% CrI was calculated using the 2.5% and 97.5% posterior quantiles.

Table 4.

Model 2’s simulated posteriors

Parameters Mean (M) Standard deviation (S) 95% CrI n_eff Rhat

Constant 8.34 0.49 (7.39, 9.31) 8844 1

gender 2.51 1.45 (-0.45, 5.40) 5980 1

agecat_gender 0.12 0.74 (-1.33, 1.62) 5929 1

Note. CrI = credible interval. The 95% CrI was calculated using the 2.5% and 97.5% posterior quantiles.

2. Narrative Interpretation of Interaction Terms While the interaction terms are now mathematically specified in the equations, their practical interpretation within the text remains somewhat dense.

• Recommendation: Please add a brief, explicit narrative in the Results section that "translates" the interaction coefficients, guiding the reader on how the effect of gender fluctuates as adolescents transition across the ordinal stages of age.

We thank the reviewer for this important suggestion. We agree that the interaction terms required clearer substantive interpretation for readers who may not be familiar with Bayesian moderation models. We have revised the Results section to provide a more explicit narrative explanation of the interaction coefficients. Please see our revisions as below:

The posterior mass for the gender coefficient was concentrated predominantly in the positive region, suggesting a positive tendency in the association between male gender and physical bullying, although the 95% CrI included zero. The posterior mass for the gender × age-category interaction term was also mainly positive, but more dispersed. Substantively, this suggests that the estimated male–female difference in physical bullying may become larger across later adolescent stages. In practical terms, male adolescents were estimated to report higher physical bullying than female adolescents, and this estimated gap appeared somewhat larger from early to middle and late adolescence. However, because the 95% CrI for the interaction term included zero, this pattern should be interpreted as a suggestive tendency rather than as strong evidence of a definitive moderation effect.

Figure 4 presents the joint posterior distribution of the gender coefficient and the gender × agecat coefficient. The posterior mass for the gender coefficient was concentrated predominantly in the positive region, suggesting a positive tendency in the association between male gender and physical victimization. By contrast, the posterior mass for the gender × age-category interaction term was much more diffuse and remained close to the weaker-effect region, indicating substantial uncertainty regarding age-based moderation. Substantively, this means that although male adolescents were estimated to report higher physical victimization than female adolescents, the estimated male–female difference in victimization did not clearly increase or decrease across early, middle, and late adolescence. Thus, unlike physical bullying perpetration, the gendered pattern of physical victimization appeared less sensitive to adolescent stage in this sample.

3. Deepening the Discussion on Selection Bias The low parental consent return rate of 24% is a critical limitation that warrants a slightly deeper critical reflection.

• Recommendation: Please expand the Limitations section by briefly discussing the potential direction of this bias. Specifically, address whether families of highly vulnerable adolescents or those with a history of bullying might have been less likely to consent, and how this self-selection might have attenuated the observed effect sizes.

We thank the reviewer for encouraging a deeper treatment of the low parental consent return rate. We agree that this is an important limitation. We have expanded the Limitations section to discuss the possible direction of participation bias more explicitly.

Second, among the 700 students who participated in the initial workshop, only 169, approximately 24%, submitted parental permission forms and were included in the final dataset. This low consent return rate raises an important risk of self-selection bias. It is possible that adolescents whose families were less engaged with school communication, had lower trust in institutional procedures, or were dealing with more sensitive bullying-related experiences may have been less likely to return parental consent forms. Families of highly vulnerable adolescents, or those whose children had a history of bullying perpetration or victimization, may also have been reluctant to participate because of stigma, discomfort, or fear of disclosure. If this occurred, the analytic sample may underrepresent students with more severe bullying involvement, thereby compressing the observed range of bullying and victimization scores and potentially attenuating the estimated associations. The findings should therefore be interpreted as patterns among consenting participants rather than as fully representative estimates for the broader student population.

4. Final Terminological Audit Despite the excellent theoretical overhaul, a few residual phrases implying biological determinism may still linger in the text.

• Recommendation: Perform a final, rigorous proofreading of the Discussion section to ensure that terms like "natural differences" are consistently replaced with construct-appropriate language, such as "socialized behavioral patterns" or "gendered expectations".

We thank the reviewer for this important caution. We agree that the manuscript should avoid language that could imply biological determinism. We therefore conducted a final terminological audit of the Discussion section and revised residual phrasing that could be read as suggesting natural or biologically fixed gender differences. In particular, we revised the title and Discussion wording to avoid framing the findings through puberty-related or biologically deterministic language.

5. Data Availability Standards

• Recommendation: Ensure that the repository details or access protocols for the secondary dataset comply strictly with PLOS ONE’s open data policy, allowing independent researchers to fully replicate the Bayesian models.

We thank the reviewer for raising this important issue. We agree that the Data Availability Statement should provide sufficient information for independent researchers to replicate the Bayesian models. We have therefore revised the Data Availability Statement to clarify both the source of the secondary dataset and the availability of the analysis code.

The dataset used in this study can be accessed at https://data.mendeley.com/datasets/sxf9kkb6t2/3. In addition, to support reproducibility, we have uploaded the R analysis script as a supplementary file.

Attachment Submitted filename: 20260609 Plos ONE editor letter.docx

# Editor report: Decision Letter 2

**DOI:** 10.1371/journal.pone.0352450.r005

**Authors:** Paul Obeng, Javier Fagundo-Rivera, Javier Fagundo-Rivera

10 Jun 2026

How Gender Is Associated with Physical Bullying and Victimization Across Adolescent Stages

PONE-D-25-51078R2

Dear Dr. Jin,

We’re pleased to inform you that your manuscript has been judged scientifically suitable for publication and will be formally accepted for publication once it meets all outstanding technical requirements.

Within one week, you’ll receive an e-mail detailing the required amendments. When these have been addressed, you’ll receive a formal acceptance letter and your manuscript will be scheduled for publication.

An invoice will be generated when your article is formally accepted. Please note, if your institution has a publishing partnership with PLOS and your article meets the relevant criteria, all or part of your publication costs will be covered. Please make sure your user information is up-to-date by logging into Editorial Manager at Editorial Manager® and clicking the ‘Update My Information' link at the top of the page. For questions related to billing, please contact billing support.

If your institution or institutions have a press office, please notify them about your upcoming paper to help maximize its impact. If they’ll be preparing press materials, please inform our press team as soon as possible -- no later than 48 hours after receiving the formal acceptance. Your manuscript will remain under strict press embargo until 2 pm Eastern Time on the date of publication. For more information, please contact onepress@plos.org.

Kind regards,

Javier Fagundo-Rivera, PhD

Academic Editor

PLOS One

**Additional Editor Comments:**

Dear Authors,

We are pleased to inform you that your manuscript PONE-D-25-51078R2: "How Gender Is Associated with Physical Bullying and Victimization Across Adolescent Stages" has been accepted for publication in PLOS ONE.

The editors and reviewers have carefully evaluated your revised submission and are satisfied that the manuscript meets the journal's standards for publication. We appreciate your thoughtful responses to the reviewers' comments and the revisions made throughout the review process.

Your manuscript will now proceed to the production stage. You will receive further communications regarding copyediting, proof review, and publication scheduling from the PLOS ONE production team.

Thank you for choosing PLOS ONE for the publication of your work. We look forward to seeing your article published and contributing to the scientific literature.

Congratulations on the acceptance of your manuscript.

Sincerely,

Javier Fagundo-Rivera, PhD

Academic Editor

PLOS ONE

Reviewers' comments: -

# Editor report: Acceptance letter

**DOI:** 10.1371/journal.pone.0352450.r006

**Authors:** Paul Obeng, Javier Fagundo-Rivera, Javier Fagundo-Rivera

PONE-D-25-51078R2

PLOS One

Dear Dr. Jin,

I'm pleased to inform you that your manuscript has been deemed suitable for publication in PLOS One. Congratulations! Your manuscript is now being handed over to our production team.

At this stage, our production department will prepare your paper for publication. This includes ensuring the following:

* All references, tables, and figures are properly cited

* All relevant supporting information is included in the manuscript submission,

* There are no issues that prevent the paper from being properly typeset

You will receive further instructions from the production team, including instructions on how to review your proof when it is ready. Please keep in mind that we are working through a large volume of accepted articles, so please give us a few days to review your paper and let you know the next and final steps.

Lastly, if your institution or institutions have a press office, please let them know about your upcoming paper now to help maximize its impact. If they'll be preparing press materials, please inform our press team within the next 48 hours. Your manuscript will remain under strict press embargo until 2 pm Eastern Time on the date of publication. For more information, please contact onepress@plos.org.

You will receive an invoice from PLOS for your publication fee after your manuscript has reached the completed accept phase. If you receive an email requesting payment before acceptance or for any other service, this may be a phishing scheme. Learn how to identify phishing emails and protect your accounts at https://explore.plos.org/phishing.

If we can help with anything else, please email us at customercare@plos.org.

Thank you for submitting your work to PLOS ONE and supporting open access.

Kind regards,

PLOS ONE Editorial Office Staff

on behalf of

Dr. Javier Fagundo-Rivera

Academic Editor

PLOS One
